# Rapid Cellular Perception of Gravitational Forces in Human Jurkat T Cells and Transduction into Gene Expression Regulation

**DOI:** 10.3390/ijms21020514

**Published:** 2020-01-14

**Authors:** Cora Sandra Thiel, Swantje Christoffel, Svantje Tauber, Christian Vahlensieck, Diane de Zélicourt, Liliana E. Layer, Beatrice Lauber, Jennifer Polzer, Oliver Ullrich

**Affiliations:** 1 Institute of Anatomy, Faculty of Medicine, University of Zurich, Winterthurerstrasse 190, 8057 Zurich, Switzerland; swantje.christoffel@uzh.ch (S.C.); svantje.tauber@uzh.ch (S.T.); christian.vahlensieck@uzh.ch (C.V.); liliana.layer@anatomy.uzh.ch (L.E.L.); beatrice.lauber@anatomy.uzh.ch (B.L.); jennifer.polzer@anatomy.uzh.ch (J.P.); 2 Department of Machine Design, Engineering Design and Product Development, Institute of Mechanical Engineering, Otto-von-Guericke-University Magdeburg, Universitätsplatz 2, 39106 Magdeburg, Germany; 3 Innovation Cluster Space and Aviation (UZH Space Hub), University of Zurich, Winterthurerstrasse 190, 8057 Zurich, Switzerland; diane.dezelicourt@physiol.uzh.ch; 4 The Interface Group, Institute of Physiology, Faculty of Medicine, University of Zurich, Winterthurerstrasse 190, 8057 Zurich, Switzerland; 5 Swiss National Center of Competence in Research (NCCR Kidney), University of Zurich, Winterthurerstrasse 190, 8057 Zurich, Switzerland; 6 Department of Industrial Engineering, Ernst-Abbe-Hochschule Jena, Carl-Zeiss-Promenade 2, 07745 Jena, Germany; 7 Zurich Center for Integrative Human Physiology (ZIHP), University of Zurich, Winterthurerstrasse 190, 8057 Zurich, Switzerland; 8 Space Life Sciences Laboratory (SLSL), Kennedy Space Center, 505 Odyssey Way, Exploration Park, FL 32953, USA

**Keywords:** simulated microgravity, vector-averaged gravity, 2D clinostat, immune cells, gene expression, microarray

## Abstract

Cellular processes are influenced in many ways by changes in gravitational force. In previous studies, we were able to demonstrate, in various cellular systems and research platforms that reactions and adaptation processes occur very rapidly after the onset of altered gravity. In this study we systematically compared differentially expressed gene transcript clusters (TCs) in human Jurkat T cells in microgravity provided by a suborbital ballistic rocket with vector-averaged gravity (vag) provided by a 2D clinostat. Additionally, we included 9× *g* centrifuge experiments and rigorous controls for excluding other factors of influence than gravity. We found that 11 TCs were significantly altered in 5 min of flight-induced and vector-averaged gravity. Among the annotated clusters were *G3BP1*, *KPNB1*, *NUDT3*, *SFT2D2*, and *POMK*. Our results revealed that less than 1% of all examined TCs show the same response in vag and flight-induced microgravity, while 38% of differentially regulated TCs identified during the hypergravity phase of the suborbital ballistic rocket flight could be verified with a 9× *g* ground centrifuge. In the 2D clinostat system, doing one full rotation per second, vector effects of the gravitational force are only nullified if the sensing mechanism requires 1 s or longer. Due to the fact that vag with an integration period of 1 s was not able to reproduce the results obtained in flight-induced microgravity, we conclude that the initial trigger of gene expression response to microgravity requires less than 1 s reaction time. Additionally, we discovered extensive gene expression differences caused by simple handling of the cell suspension in control experiments, which underlines the need for rigorous standardization regarding mechanical forces during cell culture experiments in general.

## 1. Introduction

Earth’s gravitational force has been a constant condition during Earth’s history and the evolution of cells and organisms [1]. The human organism reacts and partially adapts to altered gravity [2,3] in a time frame of a few hours up to several weeks [4]. The cellular response to microgravity, such as functional alterations [5,6], nucleus size, and shape [7,8,9], and RNA transcription [10,11] mostly occurs within seconds, sometimes months [12,13,14,15]. The question about the mechanism by which gravitational force is transmitted into a biological process was raised decades ago [16,17], but it was rarely addressed in experimental approaches. For the “sensing” of gravity in eukaryotes [18], cytoskeletal processes and mechanosensitive ion channels have been discussed, but the presented theories remained speculative and were not substantiated by systematic experiments. Therefore, none of the hypotheses of gravitational force sensing and transduction into a biological process in mammalian cells has been confirmed by experimental data so far.

Gravitational force (*F_G_*) is one of the four fundamental physical forces in nature, has an attracting character, and correlates linearly with an object’s mass. Newton’s law of gravity describes *F_G_* as the product of two objects’ masses (*m*_1_, *m*_2_) attracting each other, divided by the square of the distance (*r*), and scaled by the gravitational constant (*G*): *F_G_* = *G*(*m*_1_*m*_2_/*r*^2^). To calculate the gravitational force exerted on an object on Earth, this formula can be simplified by setting the distance between the center of the Earth and the object equal to the Earth’s radius (*R*_⊕_) and by combining *G*, Earth’s mass (M_⊕_), and *R*_⊕_ into the gravitational acceleration constant *g =* 9.81 *m*/*s*^2^, yielding *F_G_* = *mg*. As described by Einstein in his work on general relativity, there is no difference between the observation of a mass falling down in a steady reference system (related to as gravitational mass) and a resting mass in a reference system that is linearly accelerated towards the mass (related to as inertia mass). As consequence, the gravitational force exerted on an object is only measurable if the reference system is not accelerated in the same way, as for instance the International Space Station (ISS) in low Earth orbit. The ISS’s actual gravitational acceleration, *g*, that would be measurable if it was not accelerated, is only reduced by 8% due to the slightly greater distance to the Earth’s center [17], yet objects inside experience weightlessness relative to the station. As a sum of many effects, every platform that supplies weightlessness has a residual gravitational force, usually between 10^−3^ and 10^−6^ *g* [19,20]. The residual gravitational force can be due to vibrations caused by on-board machinery, atmospheric drag, solar radiation pressure, and gravitational attraction between the test object and the platform [21]. Depending on the mechanism that is studied, different quality levels of microgravity are required. If a platform supplies microgravity at a value below the mechanism’s threshold, it is also called functional weightlessness [22].

In weightlessness, hydrostatic pressure (as a result of weight of the water column on top of a reference point) and sedimentation of particles in a solution (as a result of gravitational force and buoyancy minus Stokes’ friction) are absent. Entropy-driven diffusion of particles resulting from temperature dependent Brownian motion is present, whereas concentration dependent (Raleigh) convection as a cause of buoyancy is absent. Therefore, mixing of liquids is affected by altered gravity [17].

All these changes occur in altered gravity environment and have the potential to affect biological processes. Due to the restriction of microgravity flight platforms, ground based facilities (GBFs) have been developed to simulate microgravity conditions in biological experiments [20].

Since all GBFs are ground based, none of them is able to provide true force-free conditions. By averaging forces, those devices attempt to simulate microgravity. Due to the logistical and cost advantages, the popularity of GBFs is high, although the validity of GBFs in terms of producing results comparable to flight-induced microgravity platforms remains uncertain. Clinostats rely on the principle of constantly turning a sample, thereby averaging out the gravitational force vector around one (1D/2D clinostat) or two (3D clinostat) axes. Technically speaking, the gravitational force still acts on the sample, but the direction of the gravitational vector on the sample is changing constantly. This is why the terminology "vector-averaged gravity" (vag) is usually preferred over "simulated microgravity” [23]. Whether the achieved results of a system compare to its behavior in microgravity depends on the particular effect of interest [24]. The first 2D clinostats were used to study plant gravitropism [25], in which a rotation speed of several seconds per revolution is sufficient to simulate force-free conditions [26]. In general, rotation induces centrifugal forces that act like additional gravitational forces and depend on the radius. Thus, fast rotating clinostats for cell culture experiments have a very small diameter that minimizes the distance from the center to the sample, usually in the range of millimeters [20]. Nevertheless, in addition to the centrifugal forces, Coriolis force is also acting [22]. Further, 3D clinostats are a more recent development whereby the system can independently rotate around a second axis, therefore allowing access to all three dimensions. This allows averaging forces in all directions, potentially further decreasing effects of gravitational sensing [27]. Random positioning machines (RPMs) are 3D clinostats that randomly change rotation speed and direction, which is supposed to further randomize the gravity vector possibly beneficial for averaging out gravitational effects [28,29]. However, the random movement changes seem to induce shear stress into samples [30]. Rotating wall vessels (RWVs) are devices with liquids in horizontal rotation where the speed is tuned to match the sedimentation speed of the contained objects, for example cells [31]. As consequence, cells experience a constant mode of falling downwards (sedimentation) with shear forces induced by the friction between the liquid and the objects [32]. Since sedimentation is limited by this friction, cells still experience a (reduced) gravitational force. Because an averaging of orientation may not be achieved for particles with anisotropic mass distribution, RWVs are only suitable for certain types of applications, but not for achieving vag in cellular liquid cultures. The stable levitation of diamagnetic samples like aqueous samples, and therefore most biological materials, allows true microgravity simulation, since the gravitational force is compensated for by generating a counteracting force in every point of an object and not only on the surface like for example in an RWV, preventing any internal force [33]. However, the required strong magnetic fields of several Tesla are likely to heavily influence cellular processes [34]. The wide use of clinostat microgravity simulators is based on the assumption that most biological processes have longer integration times than the period of gravitational vector rotation (e.g., 1 s for 60 rpm 2D clinorotation). The faster the studied mechanism acts, the higher the rotation frequency must be set to guarantee conditions in which the gravitational force vector is evenly distributed in all directions. Since the discovery of ultrafast reactions to altered gravity, there is debate on the effectiveness of this approach [35].

Several mechanisms induced in microgravity are also sensitive to hypergravity [3,4]. Fixed-angle centrifuges are well established and affordable platforms to generate hypergravity and 1× *g* control conditions in microgravity experiments [36]. The underlying physical principle is that objects on a circular trajectory experience centrifugal (pseudo-)force (because the rotating frame of reference is no physical inertial frame). This force (*F*_Z f_) is dependent on the rotation angular frequency (ω = 2π *f*), the radius (*r*) from the center, and the object’s mass (*m*): *F*_Z f_ = *m*ω^2^*r* = *m* 2π *f r*. With known dimensions of the centrifuge the (simulated) gravitational force can be tuned by varying the rotation frequency. Centrifuge setups with adherent cells are prone to shear forces, if the geometry of the vessel where cells are rotated in does not match the rotation circle (for example for flat bottom vessels) [17,37]. Additionally, during centrifugation, Coriolis force occurs: If an object moves linearly in an axis perpendicular to the rotating axis, the reference system (test tube/dish) moves a bit further. From the rotating reference system’s point of view, moving objects therefore exhibit a curved trajectory. The force is defined $\vec{F_{C}}=-2m(\vec{\omega }\times \vec{v})$ with *m* being the object’s mass, *ω* = 2*π f* being the centrifuge’s rotation angular frequency and *v* being the object’s speed. In a liquid, molecules and particles move around freely which is described by Brownian motion and therefore being influenced by the Coriolis force. As a consequence, particles diffuse much slower during ultracentrifugation and show an elliptic and no more ball-shaped diffusion pattern [38]. This effect is smaller by many orders of magnitude for slow rotation, e.g., when simulating slightly elevated gravitation levels, but could play a role for biological macromolecules, during cellular signaling. Therefore, centrifuges are not an ideal simulation of hypergravity. However, compared to ground based “simulators” for microgravity, this platform is much closer to the real conditions.

Although several types of cultured cells are sensitive to gravity [39,40], the immune system belongs to the most affected systems during spaceflight (reviewed in [41,42,43]). Sensitivity of human immune cells to reduced gravity has been confirmed in numerous studies in flight-induced and simulated microgravity (reviewed in [11,43]). Immune system weakening during long-term space flights could contribute to an increased susceptibility to infections, autoimmunity, and cancer during exploration class missions. Thus, it is indispensable to understand the cellular and molecular mechanisms by which altered gravity changes genomic stability and gene regulation in cells of the immune system and to assess mechanisms for adaptation. Changes of the gravitational environment induce strong alterations of human physiological systems, which respond and adapt within hours and weeks [44]. At the cellular level, changes of the gravitational force affect morphology, proliferation, differentiation, signal transduction, and gene expression [45] and have been detected within seconds in isolated cells of the immune system [10,12,13,46,47]. We recently investigated the dynamics of gene expression response to different gravitational environments in human Jurkat T lymphocytes in combination of parabolic flights with a suborbital ballistic rocket experiment and with control experiments for excluding all possible other factors of influence [13,14]. In these experiments, we detected a significantly high number of differentially regulated transcripts after 20 s of microgravity exposure [13], and gene clusters which are stable in different gravity environments [14]. According a recent comparison, the biological responses of cells in suspension in a 2D clinostat is similar to those in flight-induced microgravity [20]. We therefore aimed to validate this current state of the art opinion by systematically comparing transcriptomics data from human Jurkat T cells in microgravity provided by a suborbital ballistic rocket mission with vag provided by a 2D clinostat and hypergravity provided by 9× *g* centrifuge experiments. Thus, we performed ground based studies in a 2D clinostat and a hypergravity centrifuge using comparable experimental conditions to the parabolic flight and suborbital ballistic rocket experiments. Our aim was also to investigate if the underlying mechanisms for the perception and transduction of the gravitational force into chromatin is faster or slower than the time needed in a 2D clinostat to average gravitational force in all directions once, meaning one revolution per second. If the transcriptome effect of flight-induced microgravity can be reproduced in a 2D clinostat, the process of primary gravity perception must take longer than 1 s.

## 2. Results

### 2.1. 2D Clinostat, Centrifuge and Suborbital Ballistic Rocket Experiments with Human Jurkat T Cells

**Figure 1:**
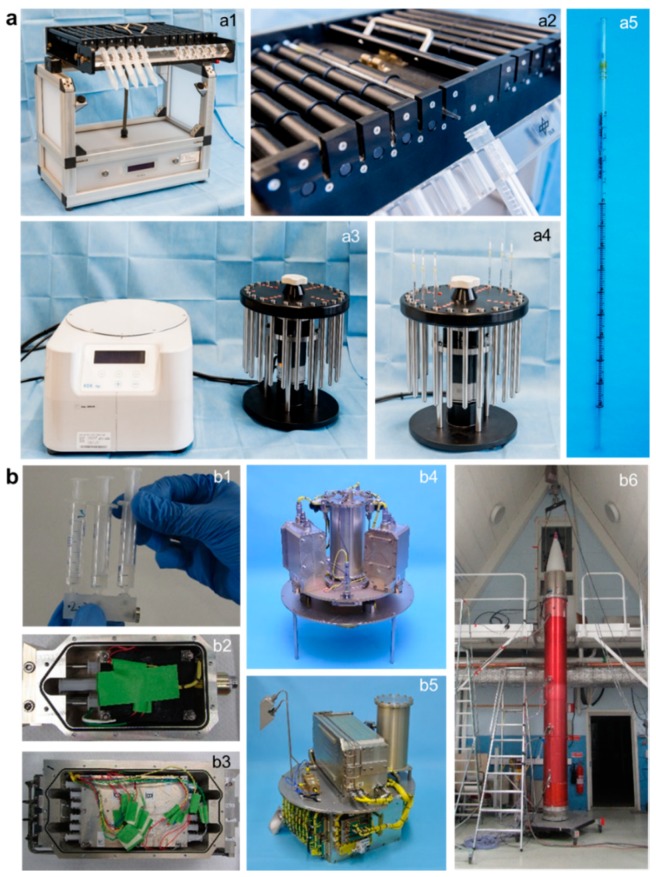
Experiment hardware used for (**a**) ground based facilities (GBFs) experiments and (**b**) experiments on board the suborbital ballistic rocket TEXUS-51. (**a1** and **a2**): Fast rotating 2D clinostat for use with 1mL pipettes built by the German Aerospace Center (DLR). (**a3** and **a4**): 9× *g* centrifuge for use with 1mL pipettes built by KEK GmbH, Bad Schmiedeberg, Germany. (**a5**): Sterile 1mL pipette (3.5 mm diameter) in which Jurkat T cells were filled and placed in the 2D clinostat or the 9× *g* centrifuge. (**b1**): For the suborbital ballistic rocket experiment Jurkat T cells, medium, and lysis buffer were filled in three syringes connected by a T piece. These assemblies were stacked in tempered and vacuum-tight containers (**b2** and **b3**) which allow hydraulic impression of the syringe plungers ensuring mixture of the fluids at defined times during flight. Containers were installed either on the static (**b5**) or the centrifuge (**b4**) position of the in-flight experiment system. (**b6**) Assembled payload structures (red) and service module (white) of the TEXUS-51 rocket. Rocket motors are not shown.

GBFs, like 2D clinostats and centrifuges, are commonly used platforms for pre-testing and verification of real microgravity (µg) and hypergravity (hyp-g) experiments. Due to the ballistic flight trajectory, it is an inherent limitation of the parabolic flight and suborbital ballistic rocket microgravity platforms that the launch and hypergravity phase is preceding the microgravity phase. The launch phase of TEXUS-51 consisted of two phases: the first one (stage 1 motor) had a duration of 12.2 s with a thrust acceleration of 5.1× *g* average and 8.1× *g* peak, and the second one (stage 2 motor) had a duration of 28.2 s and a thrust acceleration of 6.7× *g* average and 12.6× *g* peak, with an additional spin of 2.8 Hz. The pull-up-phase of the parabolic trajectory of the Airbus A310 ZERO consisted of 22s hypergravity up to 1.8× g. For this reason, we included different types of control experiments (Figure 1).

**Figure 2:**
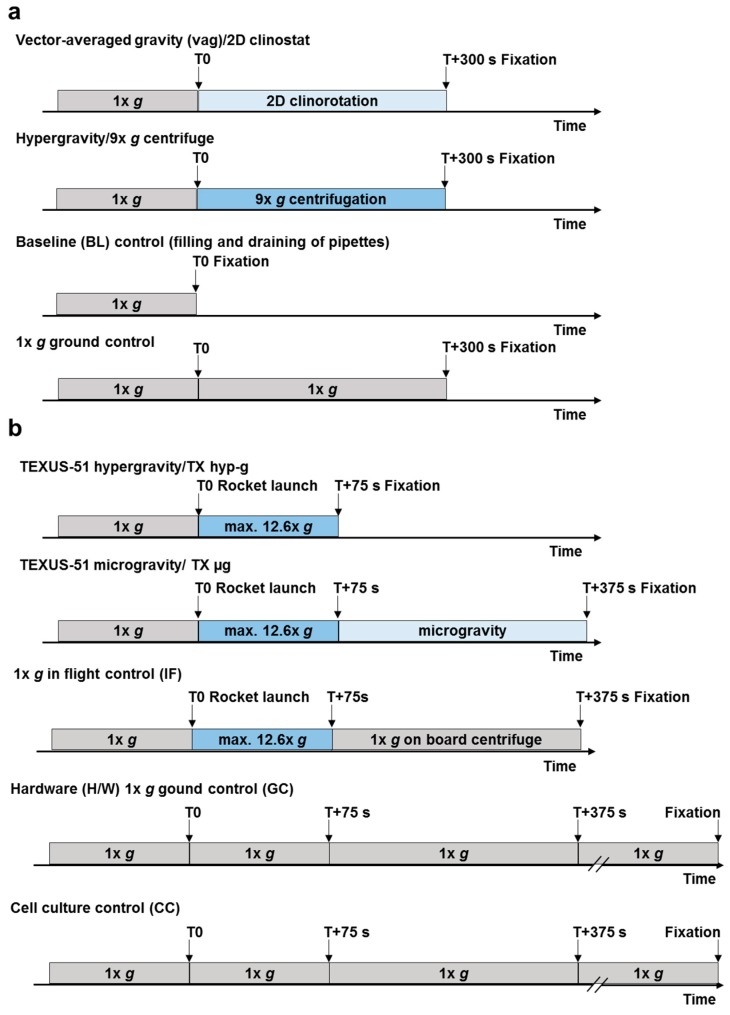
Sample lysis-scheme of ground based facilities (GBF) experiments and experiments on board a suborbital ballistic rocket flight. (**a**) During GBF experiments Jurkat T cells were exposed to 2D clinorotation or 9× *g* centrifugation. Including controls, four sample groups were included in the experiment: (1) 2D clinostat samples which were clinorotated for 300 s, (2) 9× *g* centrifuge samples which were centrifuged for 300 s, (3) Baseline (BL) control samples were lysed after the cells had been filled into the pipettes to simulate the effects of the experiment preparation handling, and (4) Hardware 1× *g* control samples were incubated at 1× *g* in parallel to the clinorotation and the centrifugation samples. (**b**) During the DLR suborbital ballistic rocket flight campaign TEXUS-51 Jurkat T cells were exposed to hypergravity and subsequently to microgravity, in total five sample groups were produced including control groups: (1) Hypergravity (TX hyp-g) samples were lysed directly after the hypergravity phase at T+75 s, (2) Microgravity (µg) samples were lysed after the 300 s microgravity phase at T+375 s, (3) 1× *g* in-flight control (1× *g* IF) samples were installed on a 1× *g* centrifuge on board the rocket and were lysed at the same time as the µg samples, (4) Hardware 1× *g* control (H/W 1× *g* GC) samples were subjected to an identical hardware on ground and were lysed about 15 min after launch, and (5) Cell culture controls (CC) were kept under standard cell culture conditions (37 °C and 5% CO_2_ in an incubator) and lysed in parallel to the H/W 1× *g* GC samples.

During the suborbital rocket experiment TEXUS-51, the following cell samples were obtained: (1) TX hyp-g: 75 s after launch, i.e. after the hypergravity phase and before the microgravity phase, (2) TX µg: after 5 min of microgravity, (3) H/W 1× *g* GC: 1× *g* on ground in the experiment hardware under identical conditions as in the rocket except the gravitational force, (4) 1× *g* IF: 1× *g* in-flight during the 5 min microgravity phase using an on-board centrifuge, and (5) CC: under regular cell culture conditions at 1× *g* to control the hardware effect (Table 1, Figure 1 and Figure 2). In the case of the GBF experiments, human Jurkat T cells were filled into sterile plastic pipettes with a diameter of approximately 3.5 mm, sealed, and inserted in the clinostat and rotated at 60 rpm for 5 min at 37 °C. Hardware 1× *g* control samples (1× *g* control) were prepared similarly and placed on the base plate of the clinostat to monitor instrument effects such as vibrations. A further 1× *g* control (Baseline; BL) was prepared, in which cells were aspirated into the pipette and immediately drained again to monitor potential effects based on the pipetting procedure. GBF hypergravity experiments were performed by installing pipettes filled with human Jurkat T cells in a centrifuge and rotated at 9× *g* for 5 min at 37 °C (Table 1, Figure 1 and Figure 2). The chosen speed of 9× *g* was in the range of the acceleration of the TEXUS-51 suborbital ballistic rocket (between 5.1× *g* for first stage mean thrust acceleration and 12.6× *g* for second stage peak thrust acceleration). For both experiment platforms, total RNA from at least five samples was processed for each group and hybridized on microarray chips.

### 2.2. Identification of Differential Gene Expression in Ground Based Facilities Experiments

**Figure 3:**
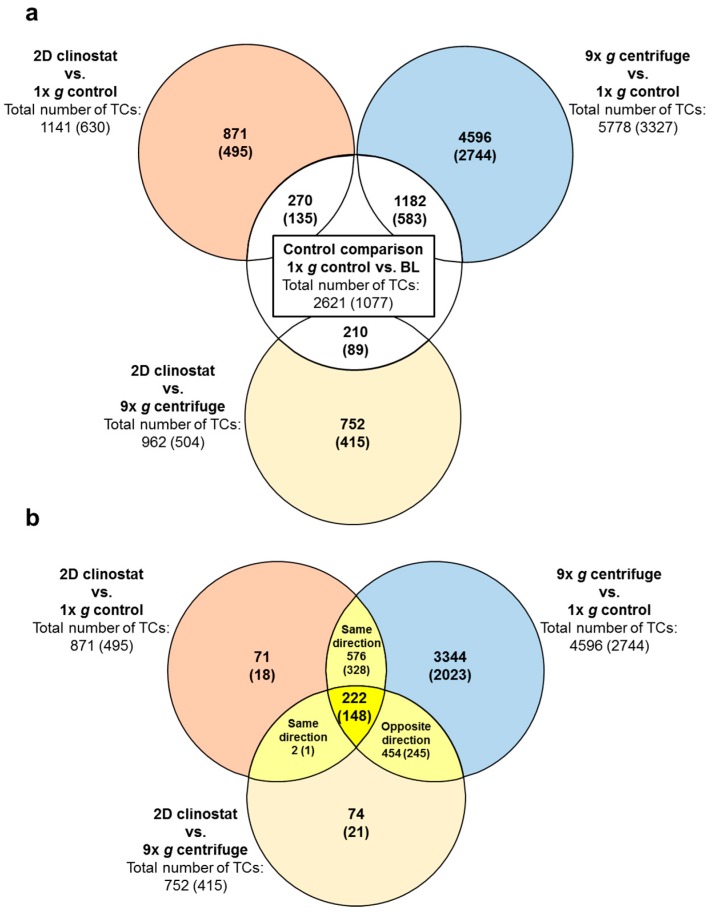
Numbers of differentially expressed transcript clusters (TCs) in comparisons of different gravitational conditions from ground based facilities (GBFs) experiments. Differential expression is defined as *p*-value < 0.05; fold change ≤ −1.3 or ≥ 1.3. Numbers are differentially expressed TCs considering all TCs on the Affymetrix GeneChip™ Human Transcriptome Array 2.0, while the numbers of differentially expressed annotated TCs are given in brackets. The Venn diagrams identify overlaps of differentially expressed TCs in the different comparisons. (**a**) Application of control: Overlaps of the comparisons of the three experimental conditions (outer circles) to the essential control comparison 1× *g* control vs. BL identifying transcripts that are sensitive to the experiment procedure itself, regardless of the gravity condition. Consequently, the TCs in the overlaps are excluded from the sets of TCs of the three experimental comparisons. The remaining sets of TCs in these experiment comparisons (colored) are therefore referred to as “1× *g* control vs. BL-controlled” or abbreviated “controlled”. (**b**) Overlaps of the controlled sets of TCs of the three comparisons. The Venn diagram identifies 222 (148) TCs that are differentially expressed in all three comparisons (yellow).

During 2D clinorotation for 5 min, 1141 TCs were differentially expressed, while during centrifugation at 9× *g* for the same time period 5778 TCs, i.e., more than five-fold, were identified to be differentially regulated. In the direct comparison of the two ground based facilities (2D clinostat vs. 9× *g* centrifuge), 962 TCs were differentially expressed (Table 2, Figure 3a). After elimination of the control comparison transcripts (1× *g* control vs. BL), in order to exclude hardware and vibration induced gene expression differences, 871 TCs and 4596 TCs were identified to be differentially expressed during clinorotation (2D clinostat vs. 1× *g* control) and 9× *g* centrifugation (9× *g* centrifuge vs. 1× *g* control), respectively (Figure 3a, Table 3). Average fold change values for vector-averaged gravity sensitive TCs (2D clinostat vs. 1× *g* control) were −1.38 and +1.39 with minimum and maximum values of −2.22 and + 2.38, respectively. Hypergravity sensitive TCs (9× *g* centrifuge vs. 1× *g* control) showed average fold change numbers of −1.44 and +1.52 with minimum and maximum values of −4.00 and +4.28, respectively. The direct comparison of 2D clinostat with 9× *g* centrifuge revealed 752 differentially expressed TCs (after subtraction of control) with average fold change values of −1.40 and +1.41 with minimum and maximum of −2.12 and +2.43, respectively (Table 3).

We further screened the data for double-sensitive transcripts in the comparisons 2D clinostat versus 1× *g* control and 9× *g* centrifuge versus 1× *g* control and identified 798 differentially regulated TCs. Hereof, 222 TCs were also differentially expressed in the comparison 2D clinostat versus 9× *g* centrifuge (Figure 3b, Table 3). The respective average, minimum, and maximum fold change expression values are shown in Table 3.

### 2.3. Comparison of GBFs with TEXUS-51 Gene Expression Results

The results of the GBFs experiments were compared to the results of the TEXUS-51 suborbital rocket experiment in order to evaluate the validity of the GBFs in preliminary studies to test experiment designs and pre-select experiment parameters for later suborbital rocket missions. For this purpose, the flight-induced microgravity-sensitive (differentially expressed in the comparison TX µg versus TX 1× *g* IF) and hypergravity-sensitive (differentially expressed in the comparison TX hyp-g versus TX 1× *g* IF) transcripts identified in the TEXUS-51 experiment were directly compared to the results from the GBF study.

**Figure 4:**
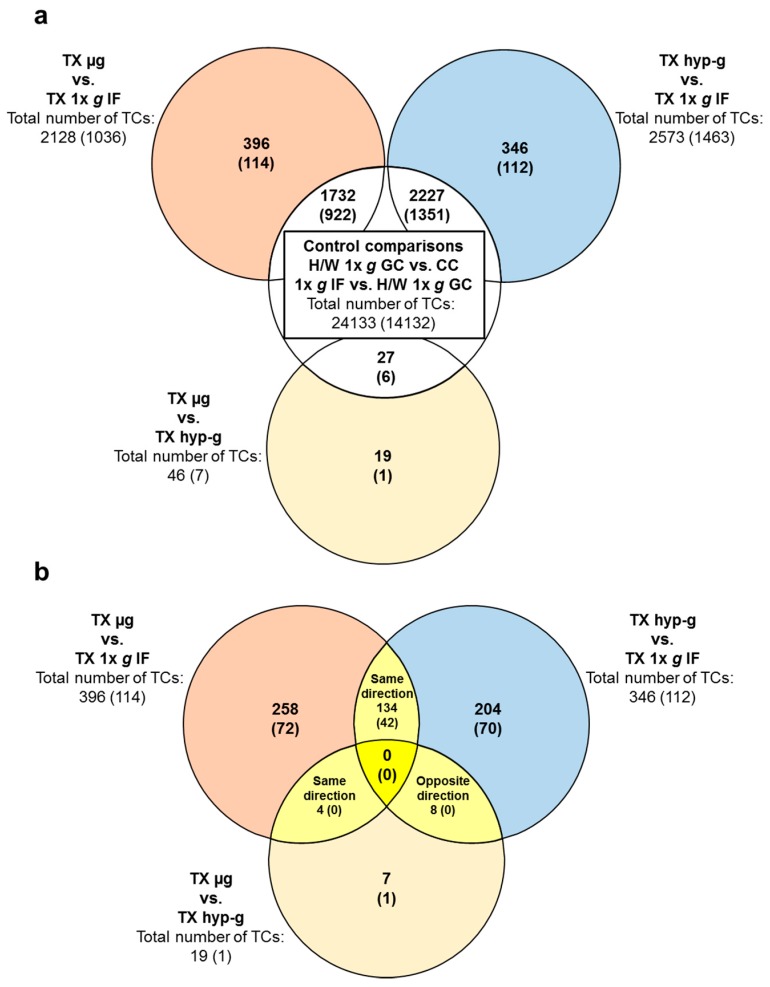
Numbers of differentially expressed transcript clusters (TCs) in comparisons of different gravitational conditions from the DLR suborbital ballistic rocket flight campaign Texus-51. Differential expression is defined as *p*-value <0.05; fold change ≤ −1.3 or ≥ 1.3. Numbers are differentially expressed TCs considering all TCs on the Affymetrix GeneChip™ Human Transcriptome Array 2.0, while the numbers of differentially expressed annotated TCs are given in brackets. The Venn diagrams identify overlaps of differentially expressed TCs in the different comparisons. (**a**) Application of controls: Overlaps of the comparisons of the three experimental conditions (outer circles) to the essential two control comparisons H/W 1× *g* GC vs. CC and 1× *g* IF vs. H/W 1× *g* GC identifying transcripts that are sensitive to the experiment procedure itself, regardless of the gravity condition. Consequently, the TCs in the overlaps are excluded from the sets of TCs of the three experimental conditions. The remaining sets of TCs in these experiment comparisons (colored) are therefore referred to as “comparisons H/W 1× *g* GC vs. CC and 1× *g* IF vs. H/W 1× *g* GC -controlled” or abbreviated “controlled”. (**b**) Overlaps of the controlled sets of TCs of the three comparisons. The Venn diagram identifies zero TCs that are differentially expressed in all three comparisons (yellow). However, several TCs are differentially expressed in the intersections of two comparisons.

In total, 2128 TCs were flight-induced microgravity-sensitive and 2573 TCs were hypergravity-sensitive in the TEXUS-51 study. In addition, 46 differentially expressed TCs could be identified in the comparison TX µg versus TX hyp-g (Figure 4a, Table 4). After filtering out all TCs that showed differential expression under control comparisons to avoid potential gravity-independent false positive hits (i.e. comparisons: H/W 1× *g* GC vs. CC and 1× *g* IF vs. H/W 1× *g* GC), we were able to identify 396 microgravity-sensitive (TX µg vs. 1× *g* IF), 346 hypergravity-sensitive (TX hyp-g vs. 1× *g* IF) and 19 TCs expressed differentially in the comparison TX µg versus TX hyp-g (Figure 4a, Table 5). Average fold change values for microgravity-sensitive TCs (TX µg vs. TX 1× *g* IF) were −1.36 and +1.40 with minimum and maximum values of −1.83 and +1.85, respectively. Hypergravity-sensitive TCs (TX hyp-g vs. 1× *g* IF) showed average fold change numbers of -1.36 and +1.45 with minimum and maximum values of −1.92 and +1.94, respectively. The direct comparison TX µg versus TX hyp-g revealed average fold change values of −1.39 and +1.37 with minimum and maximum of −1.68 and +1.43, respectively (Table 5). The analysis of “double-sensitive” TCs that are both microgravity-sensitive and hypergravity-sensitive (TX µg vs. 1× *g* IF and TX hyp-g vs. 1× *g* IF) revealed 134 TC hits (Figure 4b, Table 5). The respective fold change values for average as well as for minimum and maximum expression differences are displayed in Table 5. None of these identified “double-sensitive” transcripts were differentially expressed in the TX µg versus TX hyp-g comparison (Figure 4b).

**Figure 5:**
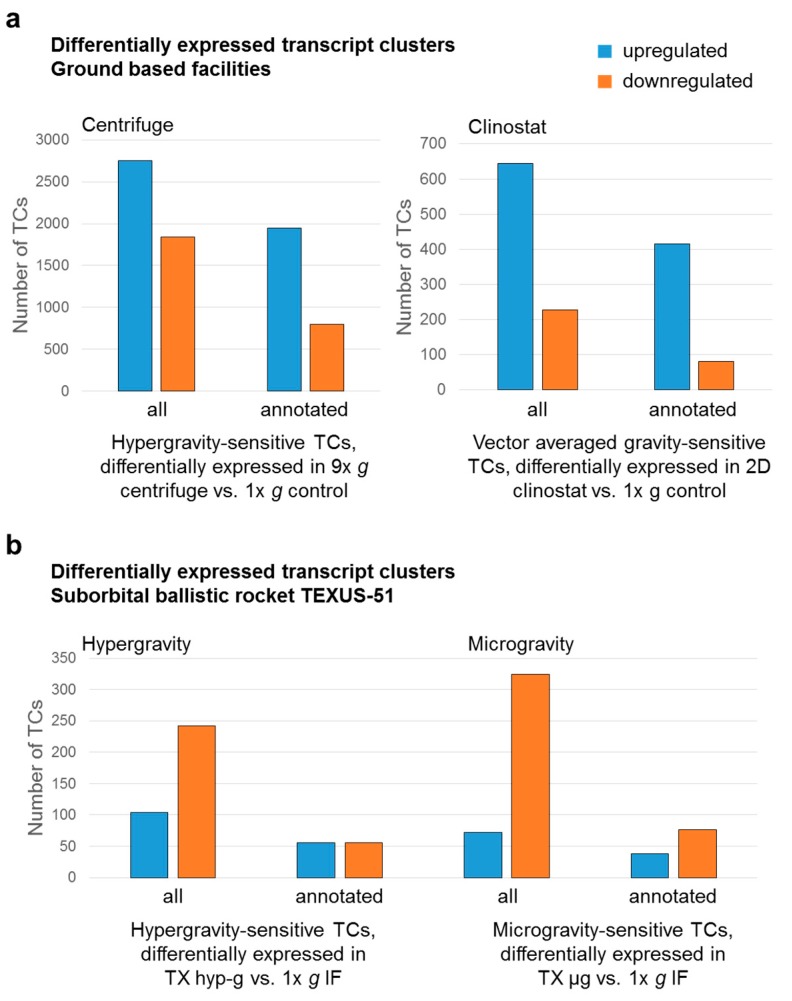
Numbers of transcript clusters (TCs) that are differentially expressed in response to hypergravity or flight-induced microgravity/vector-averaged gravity (vag). The proportion of up and downregulated TCs is shown. (**a**) Hypergravity and vag-sensitive TCs from ground based facilities experiments. (**b**) Hypergravity and microgravity sensitive TCs from suborbital ballistic rocket experiments.

Figure 5 summarizes the number of differentially expressed TCs for the GBFs and the TEXUS-51 suborbital rocket mission and divides them into up and downregulated transcripts in the different gravitational conditions. In general, in the ground based facilities clinostat and 9× *g* centrifuge, more TCs are upregulated than downregulated. Interestingly, the exposure to 9× *g* centrifugation for 5 min leads to a fivefold increase in differentially regulated transcripts compared to 5 min of 2D clinorotation. Furthermore, in hypergravity as well as in vag more transcripts are upregulated than downregulated (Figure 5a, Table 3). In contrast, the transcriptome analysis of the TEXUS-51 mission showed fewer differentially regulated transcripts in hypergravity and in microgravity. Moreover, the clear trend that more transcripts are differentially regulated in hypergravity than in microgravity cannot be observed on this platform: the total numbers of differentially regulated transcripts differ only insignificantly (396 microgravity-sensitive TCs and 346 hypergravity-sensitive TCs). Furthermore, in the dataset of the TEXUS-51 mission, the downregulated transcripts predominate in hypergravity as well as in microgravity (Figure 5b, Table 5).

**Figure 6:**
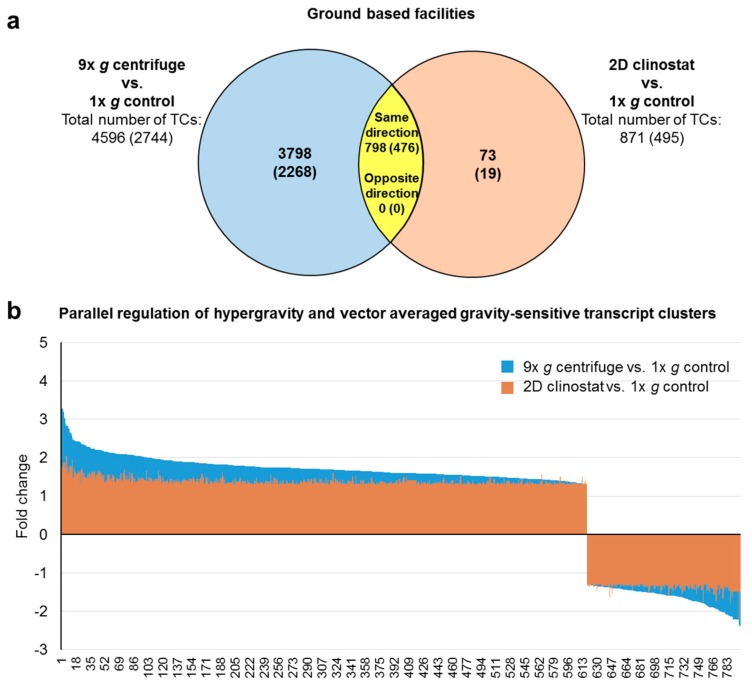
Hypergravity and vector-averaged gravity (vag) double-sensitive transcript clusters (TCs) from ground based facilities (GBFs) experiments. (**a**) Venn diagram showing overlap of hypergravity (comparison 9× *g* centrifuge vs. 1× *g* control) and vag (comparison 2D clinostat vs. 1× *g* control) -sensitive TCs (yellow area). Numbers are differentially expressed TCs considering all TC on the Affymetrix GeneChip™ Human Transcriptome Array 2.0. The numbers of differentially expressed annotated TCs are given in brackets. All TCs in the overlap section are regulated in the same direction, while none is regulated reversely. (**b**) Bar chart showing fold changes (FCs) of the 798 double sensitive TCs. FCs are ratios of the averaged expression values (linear) of a TC from the experimental groups that were compared. All TCs are regulated in the same direction in response to hypergravity and vector-averaged gravity.

**Figure 7:**
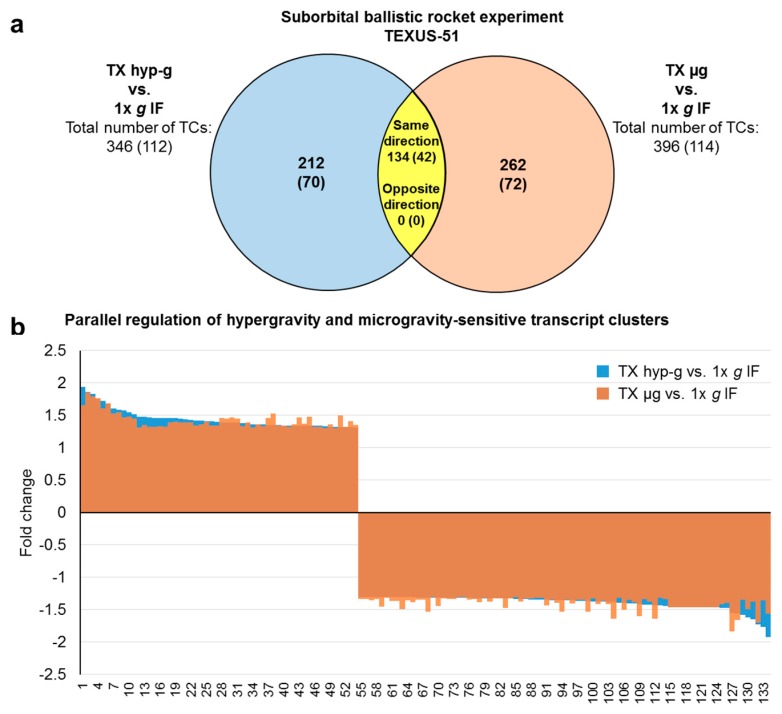
Hypergravity and microgravity double-sensitive transcript clusters (TCs) from suborbital ballistic rocket experiments. (**a**) Venn diagram showing overlap of hypergravity (comparison hyp-g vs. 1× *g* IF) and microgravity (comparison µg vs. 1× *g* IF) -sensitive TCs (yellow area). Numbers are differentially expressed TCs considering all TC on the Affymetrix GeneChip™ Human Transcriptome Array 2.0. The numbers of differentially expressed annotated TCs are given in brackets. All TCs in the overlap s are regulated in the same direction, while none is regulated reversely in hyper- and microgravity. (**b**) Bar chart showing fold changes (FCs) of the 134 double sensitive TCs. FCs are ratios of the averaged expression values (linear) of a TC from the experimental groups that were compared. All TCs are regulated in the same direction in response to hypergravity and microgravity.

Next, we performed a deeper analysis of the aforementioned “double-sensitive” TCs that react to both, hypergravity and microgravity. In the case of the GBFs experiments, 798 TCs (476 annotated TCs) could be identified which were differentially regulated in hypergravity and vag (Figure 6). This corresponds to 17% of the total number of hypergravity-sensitive and 92% of the vag-sensitive transcripts. In the TEXUS-51 suborbital rocket experiment, we identified 134 TCs (42 annotated TCs) as double-sensitive. This corresponds to 39% of the total number of the hypergravity-sensitive and 34% of the microgravity-sensitive transcripts (Figure 7). Interestingly, in the GBFs and the TEXUS-51 suborbital rocket experiment, every identified “double-sensitive” transcript was regulated consistently between hypergravity versus 1× *g* and microgravity versus 1× *g* in terms of direction of fold change (Figure 6 and Figure 7).

### 2.4. GBFs and TEXUS-51 Cross Platform Comparison of Hypergravity and Microgravity-Sensitive TCs

**Figure 8:**
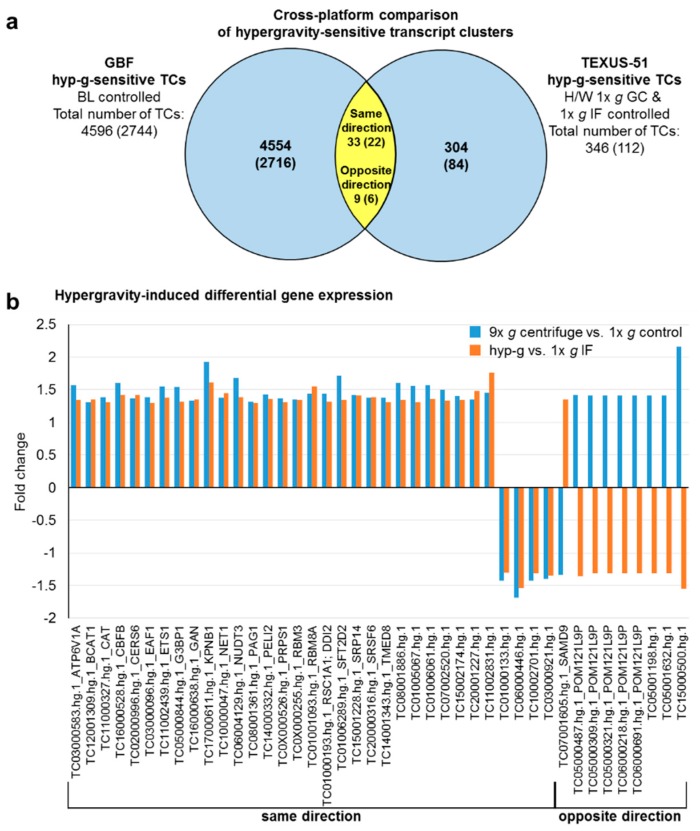
Cross platform comparison of hypergravity-sensitive transcript clusters (TCs) from ground based facilities (GBFs) and suborbital ballistic rocket experiments. (**a**) Venn diagram showing overlap of hypergravity-sensitive TCs from GBF and from suborbital ballistic rocket experiments (yellow area). Numbers are differentially expressed TCs considering all TCs on the Affymetrix GeneChip™ Human Transcriptome Array 2.0. The numbers of differentially expressed annotated TCs are given in brackets. 33 TCs in the overlap section are regulated in the same direction, while 9 are regulated reversely. (**b**) Bar chart showing fold changes (FCs) of the 42 TCs sensitive to hypergravity in both experimental platforms. FCs are ratios of the averaged expression values (linear) of a TC from the experimental groups that were compared.

**Figure 9:**
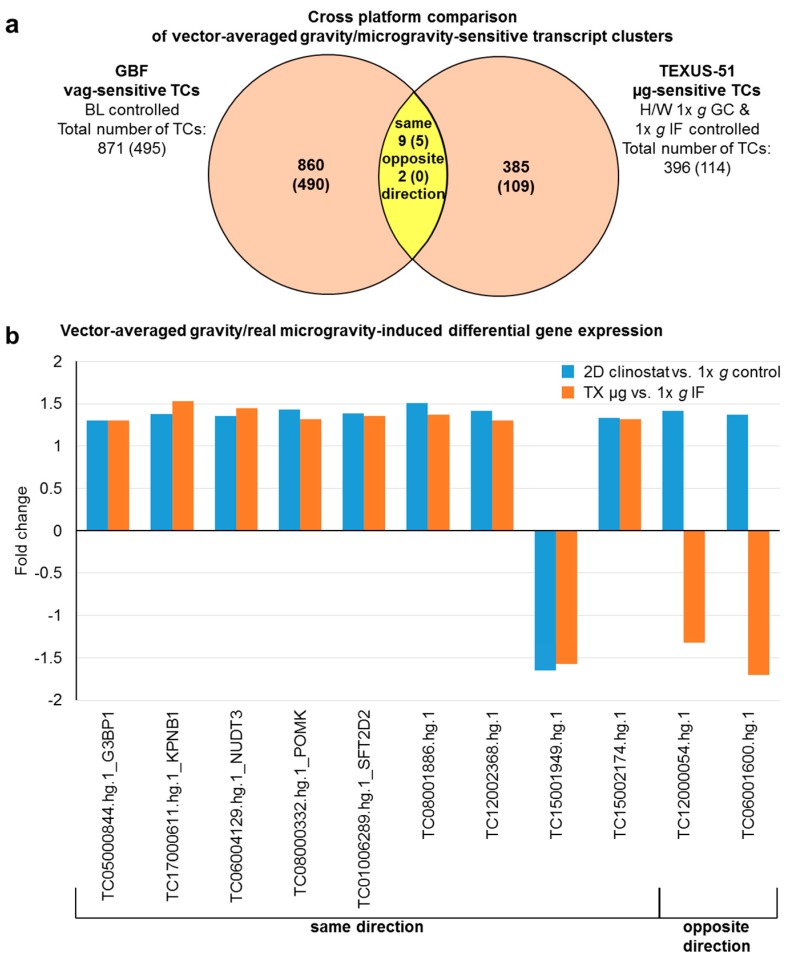
Cross platform comparison of vector-averaged gravity (vag) and flight-induced microgravity-sensitive transcript clusters (TCs) from ground based facilities (GBFs) and suborbital ballistic rocket experiments. (**a**) Venn diagram showing the overlap of the vag-sensitive TCs from GBF with the microgravity-sensitive TCs from suborbital ballistic rocket experiments (yellow area). Numbers are differentially expressed TCs considering all TC on the Affymetrix GeneChip™ Human Transcriptome Array 2.0. The numbers of differentially expressed annotated TCs are given in brackets. 9 TCs in the overlap section are regulated in the same direction, while 2 are regulated in the opposite direction. (**b**) Bar chart showing fold changes (FCs) of the 11 TCs sensitive to vag and flight-induced microgravity in both experimental platforms. FCs are ratios of the averaged expression values (linear) of a TC from the experimental groups that were compared.

**Figure 10:**
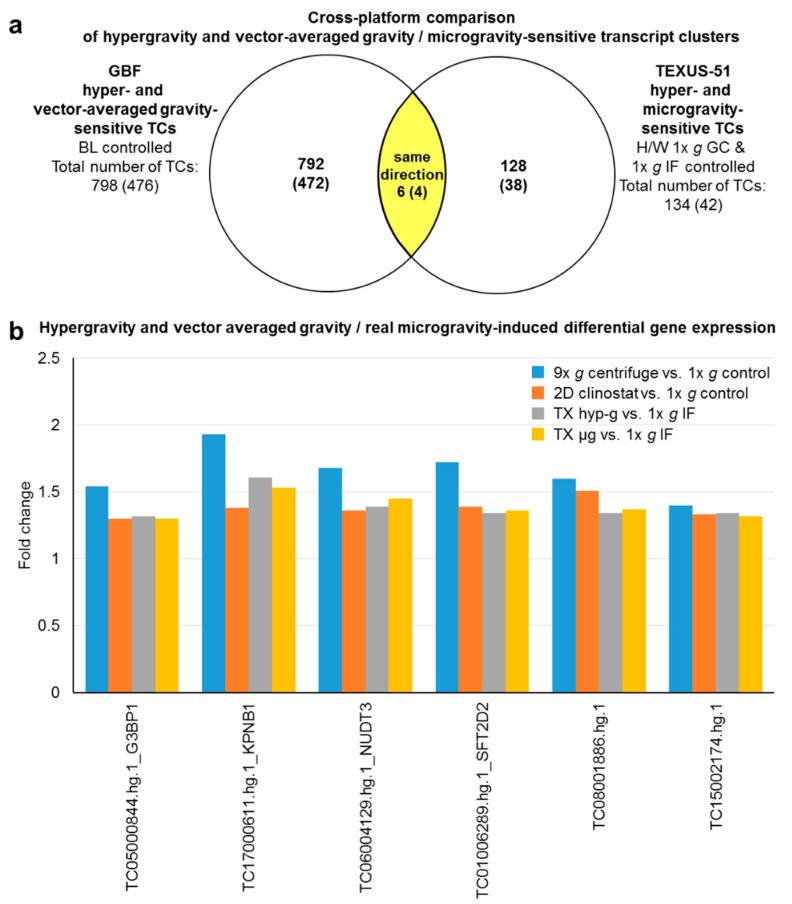
Cross platform comparison of hypergravity and vector-averaged gravity (vag)/flight-induced microgravity-sensitive transcript clusters (TCs) from ground based facilities (GBFs) and suborbital ballistic rocket experiments. (**a**) Venn diagram showing overlap of the hypergravity and vag-sensitive TCs from GBF with the hypergravity and microgravity-sensitive TCs from suborbital ballistic rocket experiments (yellow area). Numbers are differentially expressed TCs considering all TCs on the Affymetrix GeneChip™ Human Transcriptome Array 2.0. The numbers of differentially expressed annotated TCs are given in brackets. All 6 TCs in the overlap section are regulated in the same direction in all gravity conditions from both experimental platforms. (**b**) Bar chart showing fold changes (FCs) of the 6 TCs in all 4 relevant comparisons. FCs are ratios of the averaged expression values (linear) of a TC from the compared experimental groups. All TCs are upregulated in the 4 comparisons.

A cross platform comparison should benchmark how comparable the results of the GBFs experiments are with those of the TEXUS-51 suborbital flight. In case of the hypergravity-sensitive transcripts, 42 TCs (28 annotated TCs belonging to 25 genes) were identified in both platforms (Figure 8, Table 6). This represents 1% of the GBFs and 12% of the TEXUS-51 hypergravity-sensitive transcripts. 22 annotated TCs, i.e., 79% of these double-sensitive transcripts were regulated in the same direction, while six TCs were regulated in the opposite direction. Functional annotation analysis of these 25 genes identified significant enrichment in eight GeneOntology (GO) terms: transport, cytosol, nucleotide binding, poly(A) RNA binding, nuclear speck, RNA binding, intracellular membrane-bounded organelle, and regulation of alternative mRNA splicing, via spliceosome, as listed in Table 7. We also compared the intersection of vag (GBFs) and flight-induced microgravity (TEXUS-51)-sensitive transcripts. In total, 11 TCs could be identified in the intersection. This represents 1% of the GBFs and 3% of the TEXUS-51 vag/flight-induced microgravity-sensitive transcripts. 82% (five annotated TCs) of these double-positive transcripts were regulated in the same direction and none of the annotated TCs was regulated reversely (Figure 9, Table 6). Finally, we compared the hyper- and microgravity double sensitive TCs of the two platforms. Of the 798 TCs (GBFs) and 134 TCs (TEXUS-51), six TCs (four annotated TCs) could be identified which were differentially expressed in both platforms and regulated in the same direction. This represents 1% of the GBFs and 4% of the TEXUS-51 hypergravity and vector-averaged gravity/flight-induced microgravity double-sensitive TCs (Figure 10, Table 6). The functional GeneOntology based analyses revealed no significant enrichment of differentially expressed transcripts in GO terms for microgravity-sensitive and hyper- and microgravity double-sensitive TCs of the two platforms.

### 2.5. Maximally, Minimally and Non-Controlled Cross Platform Comparisons

**Figure 11:**
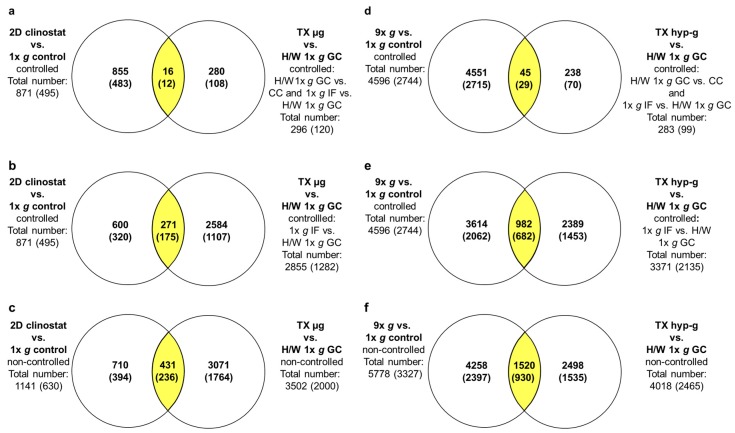
Overlap of vector-averaged gravity (vag)/flight-induced microgravity-sensitive and hypergravity-sensitive TCs from ground based facilities (GBFs) experiments and suborbital ballistic rocket flights. For the suborbital ballistic rocket experiments microgravity-sensitive and hypergravity-sensitive TCs are identified by comparison of TX µg and TX hyp-g with the H/W 1× *g* GC group. Differential expression is defined as *p*-value < 0.05; fold change ≤ −1.3 or ≥ 1.3. Numbers represent differentially expressed TCs considering all TCs on the Affymetrix GeneChip™ Human Transcriptome Array 2.0. The numbers of differentially expressed annotated TCs are given in brackets. vag/flight-induced microgravity-sensitive TCs (left panel): Venn diagrams show the overlap (yellow areas) of (**a**) GBF experiments controlled by exclusions of all transcripts that are differentially expressed in the comparison 1× *g* control vs. BL, and suborbital ballistic rocket experiments controlled by exclusion of the comparisons H/W 1× *g* GC vs. CC and 1× *g* IF vs. H/W 1× *g* GC, (**b**) GBF experiments controlled by exclusion of all transcripts that are differentially expressed in the comparison 1× *g* control vs. BL, and suborbital ballistic rocket experiments controlled by exclusion of the comparison 1× *g* IF vs H/W 1× *g* GC, (**c**) non-controlled version of the microgravity-sensitive TCs. Hypergravity-sensitive TCs (right panel): Venn diagrams show the overlap (yellow areas) of (**d**) GBF experiments controlled by exclusion of all transcripts that are differentially expressed in the comparison 1× *g* control vs. BL, and suborbital ballistic rocket experiments controlled by exclusion of the comparisons H/W 1× *g* GC vs. CC and 1× *g* IF vs. H/W 1× *g* GC, € GBF experiments controlled by exclusions of all transcripts that are differentially expressed in the comparison 1× *g* control vs. BL, and suborbital ballistic rocket experiments controlled by exclusion of the comparison 1× *g* IF vs. H/W 1× *g* GC, (**f**) non-controlled version of the hypergravity-sensitive TCs.

To assess the effects of strict filtering (strictly controlled) that have been used to avoid false positive hits of differentially expressed TCs (Figure 11a), differential gene expression in vag and flight-induced microgravity was additionally examined in minimally (Figure 11b) and non-controlled comparisons (Figure 11c). While under maximum control conditions, 16 TCs differentially expressed in flight-induced microgravity and vag could be identified, while under minimally and non-controlled conditions, 271 and 431 TCs respectively emerged as being differentially expressed for both platforms. This means that, even under non-controlled conditions, only 12% of the differentially regulated TCs identified in flight-induced microgravity could be verified by the application of vag using a 2D clinostat. On the other hand, 38% of the 1141 differentially expressed TCs identified in vag show gene expression differences in flight-induced microgravity as well. Thus, the clinostat experiments identified roughly only one third of the transcripts that were actually differentially regulated in flight-induced microgravity. We performed a similar analysis for hypergravity-sensitive transcripts and analyzed maximally, minimally and non-controlled comparisons that are differentially expressed in hypergravity on both platforms (Figure 11d–f). Here, we identified, under maximum control conditions (Figure 11d) 45 TCs, and under non-controlled conditions (Figure 11f) 1520 TCs in the intersection of the two platforms. This means that under non-controlled conditions, again 38% of the differentially regulated TCs identified in hypergravity on a suborbital rocket could be verified by experiments performed with a 9× *g* centrifuge. Conversely, from the 5778 differentially expressed TCs identified in hypergravity of the 9× *g* centrifuge, only 26%, roughly one quarter, were also found to be differentially regulated during the hypergravity phase of a suborbital ballistic rocket flight.

### 2.6. Development of Hypergravity-Sensitive TCs Over Time

**Figure 12:**
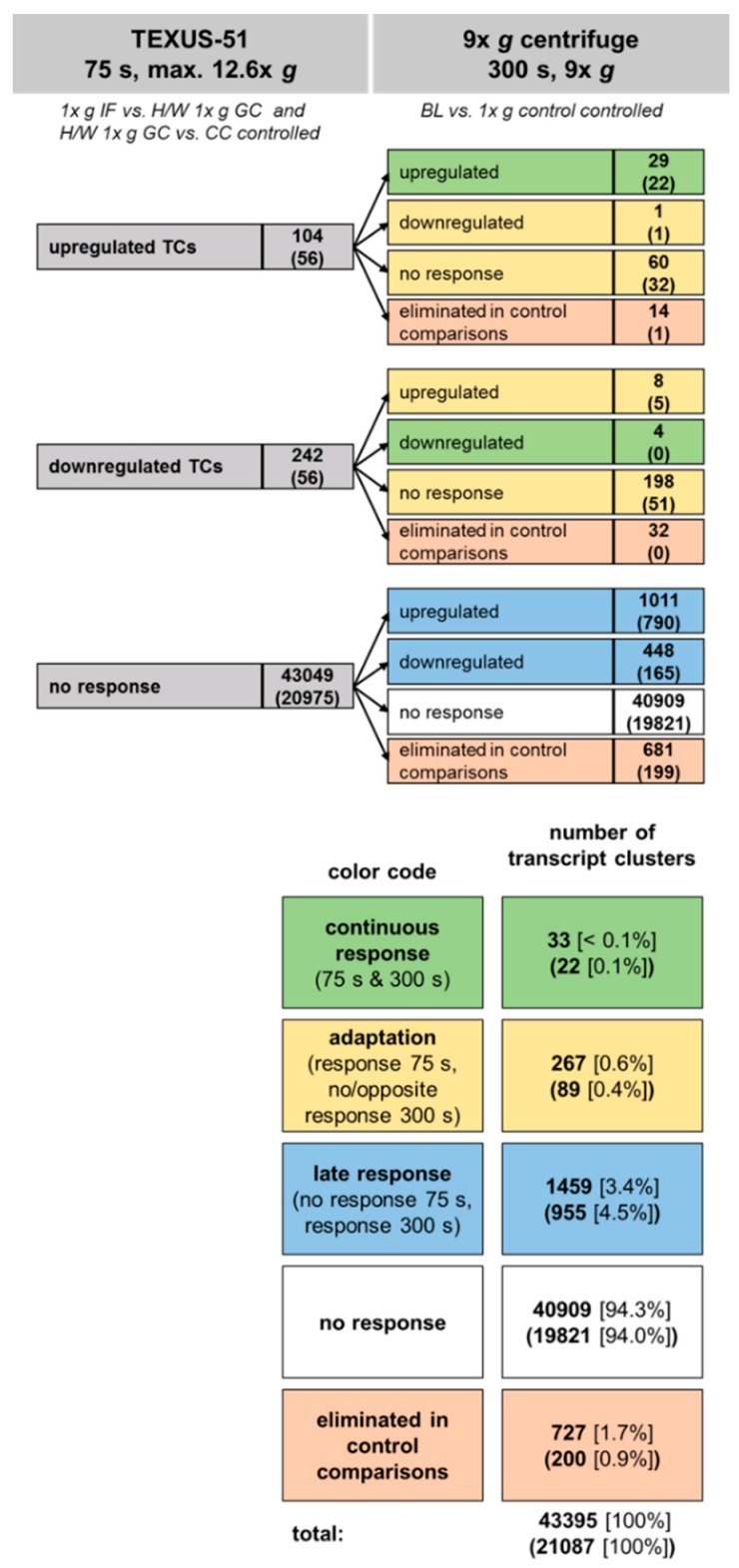
Development of hypergravity-sensitive transcript clusters (TCs) over time. Jurkat T cells were exposed to 75 s of hypergravity (max. 12.6× *g*) during the suborbital ballistic rocket flight of TEXUS-51 and to 300 s of hypergravity (9× *g*) in a centrifuge. TCs significantly up or downregulated upon hypergravity were identified by microarray-based gene expression analysis. Numbers represent differentially expressed TCs considering all TCs on the Affymetrix GeneChip™ Human Transcriptome Array 2.0. The numbers of annotated TCs are given in brackets. The TCs are grouped according to their response to 75 s of hypergravity (upper left column) and are further subdivided according to their response to 300 s of hypergravity (upper right column). Finally, the transcripts can be classified as either showing a continuous response over 75 s and 300 s hypergravity, undergoing adaption (response after 75 s but opposite or no response after 300 s), being late responsive (responsive only after 300 s), or being non-responsive to hypergravity (lower part). The vast majority of TCs did not respond to hypergravity at all. Even more TCs turned out to be adaptive (267 and 89 TCs, respectively) rather than having a continuous response (33 and 22 TCs, respectively) to hypergravity exposure.

Figure 12 summarizes the hypergravity results of the comparison of the two platforms, 9× *g* centrifuge and suborbital rocket. During the hypergravity phase of the TEXUS-51 suborbital rocket flight, 104 TCs were identified to be upregulated and 242 TCs were downregulated, while 43049 TCs showed no response. From the 104 upregulated TCs, we identified 29 TCs (28%) as also upregulated in the 9× *g* centrifuge experiment. One TC was downregulated, 60 TCs showed no response and 14 were excluded (“eliminated”) by control comparisons. In case of the 242 downregulated TCs of TEXUS-51, four TCs (2%) were also downregulated in the 9× *g* centrifuge experiment, eight TCs were upregulated, 198 TCs displayed no response, and 32 were eliminated in control comparisons. From the 43049 non-responsive TCs of the hypergravity phase of the TEXUS-51 mission, 40909 (95%) were also non-responsive in the 9× *g* centrifuge experiment. However, a large number of the non-responsive TCs identified in the TEXUS-51 platform were found to be differentially expressed in the 9× *g* centrifuge experiment (1011 upregulated and 448 downregulated).

Relative to the total number of TCs examined (43395), 33 TCs (< 0.1%) showed a continuous response to hypergravity, 267 TCs (0.6%) an adaptation, 1459 TCs (3.4%) a late response and 40909 TCs (94.3%) no response at all. Meanwhile, 727 TCs (1.7%) were eliminated in control comparisons (Figure 12).

### 2.7. Comparison of Gene Regulation in Flight-Induced Microgravity and Vector-Averaged Gravity

**Figure 13:**
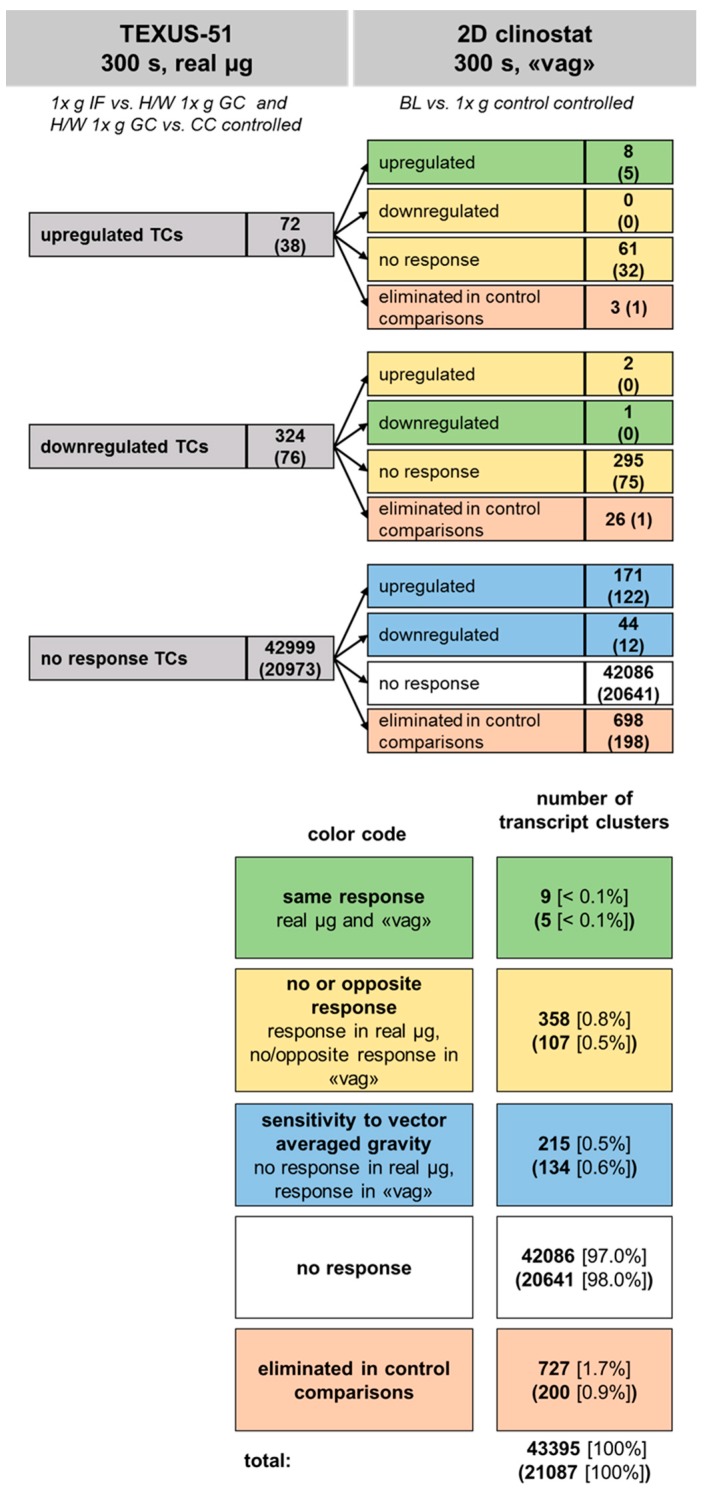
Comparison of gene regulation in flight-induced microgravity and vector-averaged gravity (vag). Jurkat T cells were exposed to 300 s of microgravity during the suborbital ballistic rocket flight of TEXUS-51 and to 300 s of vag in a 2D clinostat. Significantly up or downregulated transcript clusters (TCs) were identified by microarray-based gene expression analysis. Numbers represent differentially expressed TCs considering all TCs on the Affymetrix GeneChip™ Human Transcriptome Array 2.0. The numbers of annotated TCs are given in brackets. The TCs are grouped according to their response to flight-induced microgravity (upper left column) and are further subdivided according to their response to vag (upper right column). Finally, the transcripts can be classified as showing either the same response vag and flight-induced microgravity, no or reverse response, response to vag, or not responsive at all. The vast majority of TCs did not respond to neither type of microgravity. Even more TCs were not regulated in the same way in flight-induced microgravity as in vag, rather than showing the same response.

Figure 13 summarizes the results of the comparisons of the two platforms regarding vag and flight-induced microgravity. During the microgravity phase of the TEXUS-51 suborbital rocket flight, 72 TCs were identified to be upregulated and 324 TCs were downregulated, while 42999 TCs showed no response. From the 72 upregulated TCs, we identified eight TCs (11%) as also upregulated in the 2D clinostat experiment. No TC was downregulated, 61 TCs showed no response, and three TCs were eliminated by control comparisons. In case of the 324 downregulated TCs of TEXUS-51, one TC (< 1%) was also downregulated in the 2D clinostat experiment, two TCs were upregulated, 295 TCs displayed no response, and 26 TCs were eliminated in control comparisons. From the 42999 non-responsive TCs of the TEXUS-51 mission, 42086 (98%) were also non-responsive in the 2D clinostat experiment. However, a number of the non-responsive TCs identified for the TEXUS-51 platform were found to be differentially expressed in the 2D clinostat experiment (171 upregulated and 44 downregulated).

Relative to the total number of TCs examined (43395), nine TCs (< 0.1%) showed the same response in flight-induced and vag, 358 TCs (0.8%) showed no or reverse response, 215 TCs (0.5%) were sensitive only to vag, and 42086 TCs (97.0%) displayed no response at all. Further, 727 TCs (1.7%) were eliminated in control comparisons (Figure 13).

### 2.8. Cross Platform and Altered Gravity Condition Overlap Analysis of TC Regulation in Vector-Averaged Gravity, Flight-Induced Microgravity, and Hypergravity in GBF and TEXUS-51

Table 8 displays a cross platform comparison. In the left column, the number of upregulated, downregulated and non-responsive TCs of the primary comparison are shown. In the right part of the table, the distribution of these TCs within the remaining altered gravity comparisons is listed. In case of the primary comparison 2D clinostat versus 1× *g* control, most of the differentially regulated TCs (94.5% and 68.9%, respectively) can also be found in the comparison 9× *g* centrifuge versus 1× *g* control. For the primary comparison TX µg versus 1× *g* IF representing differential gene expression in flight-induced microgravity, only 11.6% of the upregulated and 1% of the downregulated TCs can be confirmed in vag (2D clinostat vs. 1× *g* control). Interestingly, the major part of the TCs can also be found in the hypergravity comparison 9× *g* centrifuge vs. 1× *g* control (34.8%) and TX hyp-g vs. 1× *g* IF (73.91% of the upregulated TCs and 21.81% of the downregulated TCs). Regarding the primary comparison 9× *g* centrifuge versus 1× *g* control, 16.3% of the upregulated and 6.8% of the downregulated TCs could be confirmed in the comparison 2D clinostat versus 1× *g* control. In case of the primary comparison TX hyp-g vs. 1× *g* IF, about one third (32.2%) of the upregulated TCs could be confirmed to be upregulated in the comparison 9× *g* centrifuge versus 1× *g* control. Additionally, 56.7% of the upregulated and 31.0% of the downregulated hypergravity-sensitive TCs could be verified in flight-induced microgravity, indicating that many of the identified TCs are hypergravity and microgravity “double sensitive”.

## 3. Discussion

We analyzed the influence of altered gravity on the gene expression response of non-activated Jurkat T lymphocytes using the GBFs fast rotating 2D clinostat and a 9× *g* centrifuge. Both platforms hold the same type of pipettes containing cell samples. During 5 min of vag in the clinostat, we found 768 significantly upregulated and 373 significantly downregulated TCs compared to 1× *g* controls, while 5 min of 9× *g* hypergravity induced 3046 significantly upregulated and 2732 significantly downregulated TCs. The baseline control (1× *g* control vs. BL) revealed 2125 significantly upregulated and 496 significantly downregulated TCs, reflecting the influence of filling of the cell suspension into the pipettes and releasing the cell suspension from the pipettes (Table 2). Eliminating those significantly differentially expressed TCs due to pipette usage yielded 644 upregulated and 227 downregulated TCs after 5 min of vag, and 2753 upregulated and 1843 downregulated TCs after 5 min of 9× *g* hypergravity (Table 3). The significantly differential gene expression response ranged between −4.00 and +4.28-fold changes.

We also analyzed the gene expression in non-activated human Jurkat T lymphocytes in microgravity and hypergravity during the TEXUS-51 sounding rocket campaign. The suborbital ballistic flight provided 75 s of hypergravity during the rocket launch followed by 5 min of flight-induced microgravity. Exposure of Jurkat cells to 5 min of hypergravity in the 9× *g* GBF centrifuge and to hypergravity of the rocket flight resulted in 42 differentially regulated TCs respectively, 28 annotated TCs being significantly differentially expressed using both research platforms (Figure 8 and Figure 12, Table 6). A functional gene ontology analysis of the associated genes revealed an association of the genes to transport, cytosol, nucleotide binding, Poly(a) RNA binding, nuclear speck, RNA binding, intracellular membrane-bounded organelles, and regulation of alternative mRNA splicing via the spliceosome (Table 7).

When we compared the vag -sensitive TCs of the GBF experiment with the flight-induced microgravity-sensitive TCs of the suborbital rocket flight, 11 TCs respectively (five annotated TCs) were significantly altered using both research platforms (Figure 9 and Figure 13). Among these annotated five TCs are: (1) the G3BP1 (G3BP stress granule assembly factor 1) enzyme, that unwinds DNA and RNA duplexes in an ATP-dependent manner, (2) KPNB1 (karyopherin subunit beta 1) which is involved in nucleocytoplasmic transport of e.g. ribosomal proteins or H1, H2A, H2B, H3, and H4 histones, (3) NUDT3 (nudix hydrolase 3) involved in nucleoside phosphate metabolic pathways and negatively regulating the ERK1/2 pathway, (4) POMK (protein-O-mannose kinase), which is involved in forming transmembrane linkages between the extracellular matrix and the exoskeleton, and (5) SFT2D2 which seems to participate in the fusion of retrogradely transported endosomes with the Golgi complex (Table 6 and Table 9). A functional gene ontology analysis of these TCs showed no significant association to cellular functions or processes.

The analysis of hypergravity and microgravity double-sensitive TCs in both platforms revealed four annotated TCs associated with 4 genes: (1) *G3BP1*, (2) *KPNB1*, (3) *NUDT3*, (4) *SFT2D2*, all of which have already been identified as vag/flight-induced microgravity-sensitive (Table 6).

Interestingly, the direct comparison of hypergravity and vag/flight-induced microgravity-sensitive TCs between the platforms showed that the GBF experiments revealed about five times as many hypergravity as vag-sensitive TCs. Furthermore, the 2D clinostat and 9× *g* centrifuge experiments showed more up than downregulated TCs. We could not observe this effect in the data of the TEXUS-51 mission. Here, no major differences between the number of hypergravity and flight-induced microgravity-sensitive TCs were observed, and the differentially expressed TCs were rather downregulated instead of upregulated (Figure 5).

Our results indicate that less than 1% of all examined transcripts show the same response in vag and flight-induced microgravity. Even when only considering the 396 transcripts differentially expressed in flight-induced microgravity, less than 12% show the same response in flight-induced microgravity and vag. Based on these surprising results, we have reinvestigated the forces prevailing in a 2D clinostat, commonly accepted to be classified as simulated microgravity. To simplify the calculations for our system, we assumed that human Jurkat T cells in suspension behave like small particles.

The study of particle behaviors in flows is a fundamental problem in fluid dynamics and a number of theoretical, computational, and experimental studies have investigated the trajectory of spherical particles in purely rotational flows. Given the dimensions of the cells and clinostat, only studies pertaining to low Reynolds number flows are relevant in this context. Lin and colleagues [48] theoretically analyzed the trajectories of particles in rotational flow at small but finite Reynolds numbers and demonstrated that buoyant particles converge to a stable equilibrium position located under the rotational plane in accordance with experimental observations [49]. In contrast, particles denser than the surrounding fluid do not have a stable equilibrium point and ultimately follow a divergent trajectory, spiraling outward. Brought back to the dimensions of the problem at hand, the spiraling motion of the particle over 5 min would not markedly deviate from the circular motion of the surrounding media.

In shear and rotational flows, fluid inertia further causes the particle to spin. For small Taylor numbers ($\mathrm{Ta}=\frac{r^{2}\omega }{\mu /\rho }$) (with r: radius, ω: angular velocity, µ: viscosity, p: density) the angular velocity of the particle ($\Omega_{p}$) relates to that of the fluid as follows:

$$\frac{\Omega_{p}}{\omega }=1-0.3076\cdot {\mathrm{Ta}}^{\frac{3}{2}}+o\left({\mathrm{Ta}}^{\frac{3}{2}}\right)$$

In our application, Ta is in the order of $10^{-5}$. The particle angular velocity can thus be considered equal to that of the clinostat. 

**Figure 14:**
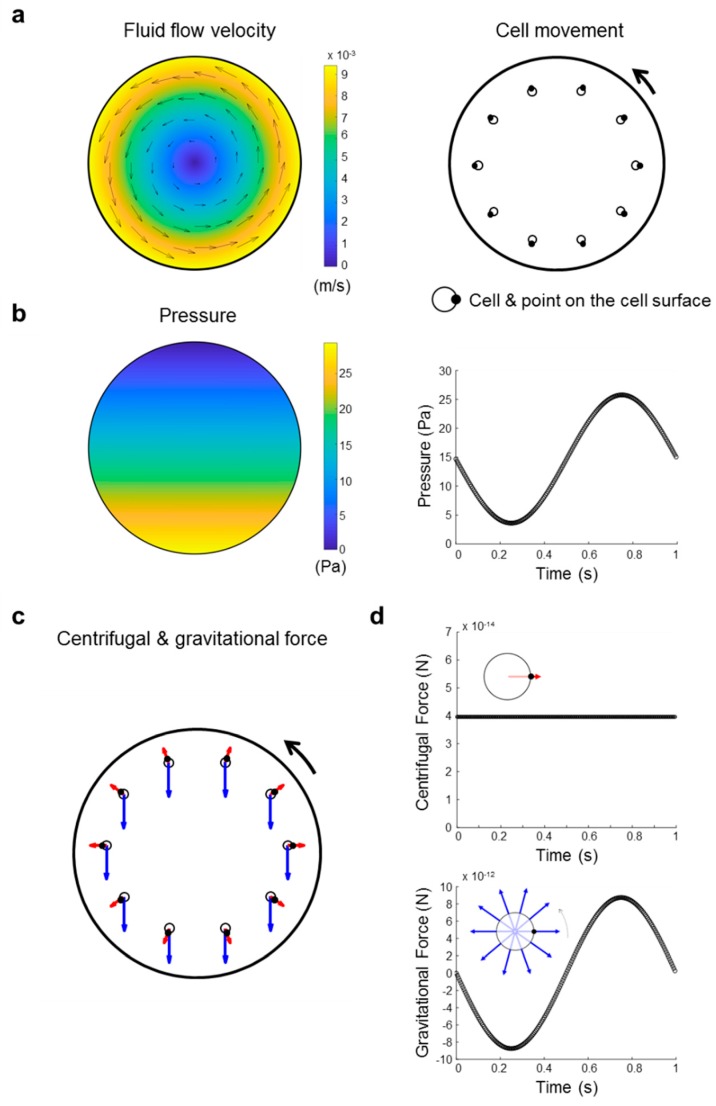
Illustration of the trajectory of a spherical particle with the same diameter and density as the investigated Jurkat T cells over one clinorotation. (**a**) The fluid flow velocity differs depending on the location in the rotating vessel. Cells in suspension follow the fluid flow in the system. The circle represents a cell with the black dot labelling a fixed location on the cell surface to help visualize the cell orientation. (**b**) Evolution of the average pressure on the cell over time. The mean pressure over 1 s is 14.7 Pa. However, at the bottom the pressure is 29.4 Pa higher compared to the top of the pipette. Gravitational (blue arrow) and centrifugal forces (red arrow) experienced at selected locations along the cell trajectory in the laboratory reference frame (**c**) and in the rotating reference frame (**d**). In the rotating reference frame, the radial component of the centrifugal force is constant while the radial component of the gravitational force is periodic. Note that for visibility on the figure, the particle diameter is scaled up 10 times relative to that of the clinostat and the centrifugal force is scaled up 100 times compared to gravity. Black arrow: direction of rotation.

Summarizing the above, Figure 14 illustrates the general motion of a spherical particle matching the characteristic dimension and density of human Jurkat T cells over one revolution of clinorotation. As particle and clinostat have the same rotation rate, the gravitational force and associated hydrostatic pressure gradients follow a cyclic pattern in the particle reference frame, for the gravitational force with a null average, and for the pressure with an average of 15 Pa over one revolution per second. In contrast, the centrifugal force and resultant hydrostatic pressure gradients, as well as shear stresses associated with the outward radial displacement of the particle, have a constant direction in the particle reference frame (Figure 14d). However, as stated earlier, it should be kept in mind that the maximal centrifugal force in the clinostat is 0.006× *g* and therefore very small compared to the normal unidirectional gravitational force.

In contrast to real microgravity environments, cells in a clinostat still experience gravitational forces albeit with a constantly changing direction (Figure 14c,d). The Jurkat T cells in our experiment are constantly exposed to oscillating forces with maximum changes of 29.4 Pa, 2.3 mPa, and 17.5 × 10^−12^ N for pressure, shear stress, and gravitational force, respectively (Figure 14). For a rotation speed of 60 rpm, the direction of gravitational forces integrated over 1 s (corresponding to one revolution of the clinostat) are averaged. According to the clinostat theory [20,30], the biological effect of gravitational forces is canceled, if the initial trigger mechanism requires at least one second of stimulation, corresponding to one full 360-degree rotation in a 2D clinostat rotating with 60 rpm. Because the transcriptome response during the microgravity phase provided by a suborbital ballistic rocket flight was not detectable in vag provided by a 2D clinostat, it is unlikely that the initial trigger of gravitational force transduction into the chromatin lasts longer than one second. 

Our experiments are limited by the inherent limitation of ballistic trajectories, the launch and hypergravity phase, which is preceding always the microgravity phase. Although we applied four types of control experiments (Figure 1), not controlled artifacts induced by the preceding force conditions cannot be fully excluded. In our experiments, only less than 1% of the transcriptome changes in flight-induced microgravity could be reproduced by clinorotation. Our data suggest that the initial trigger and perception mechanisms of gene expression changes in microgravity is faster than one second. Indeed, mechanical force transduction into the chromatin occurs within milliseconds [50], allowing the nuclear structure to respond directly without biochemical signaling [51,52]. In previous studies which have investigated the oxidative burst reaction in mammalian macrophage cells in microgravity, rapid adaption to a microgravity environment was detected in microgravity on board of the ISS [10], but not in the vag of clinorotation experiments [12,53,54].

If 99% of such pivotal events cannot be simulated by analogous clinostat experiments, the general assumption that 2D clinostats provide a valid simulation of microgravity and can be recommended for most biological organisms [20] should be revised. If in addition the primary trigger of gravity-induced gene expression response [10,46] is less than one second, the fundamental question of suitability of the clinostat system for the investigation of mammalian cells arises. We therefore recommend that sufficient validation experiments should be carried out before starting experiments with vag as simulation for real microgravity.

Additionally, we discovered extensive gene expression differences caused by simple handling of the cell suspension in control experiments, which underlines the need for rigorous standardization regarding mechanical forces occurring during cell culture experiments in general.

## 4. Materials and Methods

Material and methods have been described in our previous studies [13,14].

### 4.1. Cell Culture

The standard cell culture conditions for the human cell line Jurkat T cells (ATCC Clone E6-1, TIB-152, Manassas, VA, USA) were described previously [3,4]. Briefly, cells were kept in a standard cell culture incubator (5% CO_2_, 100% humidity, 37 °C) and were cultured in RPMI1640 medium (Biochrom/Merck Millipore, Darmstadt, Germany), supplemented with 10% fetal bovine serum (FBS Superior; Biochrom/Merck Millipore, Darmstadt, Germany), 2 mM glutamine (low endotoxin; Biochrom, Darmstadt, Germany), and 100 U/mL penicillin, as well as 100 µg/mL streptomycin (Biochrom, Darmstadt, Germany). The Jurkat cells were subcultured every other day and reseeded at a density of 0.2 × 10^6^ cells/mL. A trypan blue assay was used for the quantification and assessment of cell viability (between 97% and 100%). The measured population doubling time ranged between 24 and 28 h.

### 4.2. TEXUS-51 Suborbital Ballistic Rocket Experiment

TEXUS-51 was a German Center for Aerospace (DLR) funded suborbital ballistic rocket mission. The vehicle assembly comprised the experiment payload on top of a two-stage VSB-30 rocket motor. The rocket launch was performed on 23 April 2015 at 09:35 from the ESRANGE Space Center in Sweden. Excerpt of the flight profile: (i) altitude: 258 km, (ii) total microgravity time: 369 s (10^−5^× *g*), (iii) 8.1× *g* first stage peak thrust acceleration, (iv) 5.1× *g* mean thrust acceleration, (v) first stage burnout at 12.1 s, (vi) engine separation at 13.4 s, (vii) 12.6× *g* second stage peak thrust acceleration, (viii) 6.7× *g* mean thrust acceleration, (ix) burnout at 43.2 s, (x) yo-yo despin at 56.0 s, and (xi) motor separation at 59.0 s.

### 4.3. TEXUS-51 Mission Procedures

The TEXUS-51 mission procedures have been previously described [13,14,55,56]. Briefly, three syringes were assembled into one unit. One syringe was filled with human Jurkat T cells (25 million cells), a second syringe contained cell culture medium, and a third syringe contained TRIzol LS (Life Technologies, Darmstadt, Germany) as lysis solution. The syringes were closed by small plugs to prevent the premature mixture of the liquids. A T piece connected the three syringes of one unit which was integrated into a temperature controlled and vacuum-resistant container (Figure 1(b1)). The activation of the syringes was controlled automatically during the experiment at pre-set times using a pneumatic system. Seven hours before launch, the experiment preparation started, and integration of the experimental units took place 1:15 h to 0:45 h before launch via a late access port. During the whole experiment and until lysis, the samples were temperature controlled at 36.5 °C ± 0.5 °C. The disassembly and purification of samples were performed immediately after landing and recovery of the payload. Total RNA was kept at −80 °C until processing for the microarray analysis.

### 4.4. Experimental Preparation Procedures for TEXUS-51 Experiments

Human Jurkat T cell culture was performed as previously described [13,14,55,56] in the ESRANGE Space Center laboratory facilities. Briefly, sterile plastic syringes were filled with 0.5 mL Jurkat cells (25 million cells). Additionally, a second syringe was loaded with 0.3 mL cell culture medium, and 1 mL of TRIzol LS (Life Technologies, Darmstadt, Germany) was filled in a third syringe. These three syringes were assembled into one experimental unit with a sterile plastic T block including a connecting tubing system. The experiment units were kept at 36.5 °C ± 0.5 °C until either integration into the flight module or execution of ground controls. The experiment sequence was as follows: injection of (i) 0.3 mL cell culture medium, (ii) 1 mL TRIzol LS into the syringe containing the cells according to pre-defined time points (Figure 2). In total, 39 samples were processed during the TEXUS-51 mission: 7 × 1× *g* ground cell culture controls (CC), 7 × H/W 1× *g* GC, 9 × 1× *g* IF, 7 × BL-TX hyp-g and 9 × µg.

### 4.5. Ground Based Facilities (GBFs) Experiments

Ground based simulation of microgravity is a common technique to pre-test and prepare spaceflight experiments. In our experiments, 2D clinorotation (DLR pipette clinostat, Institute for Aerospace Medicine, Gravitational Biology Group, Cologne, Germany) and centrifugation (pipette centrifuge, KEK GmbH, Bad Schmiedeberg, Germany) were used for vag and hypergravity experiments, respectively. For vag and centrifugation experiments, human Jurkat T cells from the same cell pool were used for both experiment conditions. Then, 0.8 mL of cell suspension (25 million cells) was filled in a 1 mL pipette (Becton Dickinson/Falcon, Basel, Switzerland) corresponding to the total amount of cells per sample in the TEXUS-51 experiment. One end or both ends of the pipettes were sealed by rubber plugs depending on whether they were used for clinorotation or centrifugation. Pipettes containing Jurkat cells were then either placed in the 2D clinostat rotating with a speed of 60 rpm or in the centrifuge set to 9× *g* at 37 °C for 300 s (Figure 1). The centrifugation force of 9× *g* was chosen to simulate the acceleration of the TEXUS-51 suborbital ballistic rocket which lies in the range between 5.1× *g* (first stage mean thrust acceleration) and 12.6× *g* (second stage peak thrust acceleration). For control experiments, pipettes filled with Jurkat cells were placed on the base plate of the clinostat. These 1× *g* hardware controls were exposed to all non-clinorotation hardware effects including instrument vibration for 300 s. A fourth sample group representing a pre-experiment baseline including potential shear forces acting on the cells by aspiration and draining out of the pipette was performed to monitor the effects of the filling procedure. After the respective incubation times, cell samples were mixed with 1 mL TRIzol LS (Life Technologies, Darmstadt, Germany) and homogenized with a needle (0.8 × 80 mm; B Braun Melsungen, Melsungen, Germany) and a syringe. Total RNA was purified as described in the following Section 4.6. Per sample group, 6 samples were analyzed with an Affymetrix GeneChip™ Human Transcriptome Array 2.0.

### 4.6. RNA Purification

A detailed protocol has been published previously [13,14,55,56]. In summary, the total sample volume of 1.8 mL was sheared three times by passing through a 20 G needle (B. Braun Melsungen, Melsungen, Germany). Subsequently, 0.2 mL chloroform (Sigma-Aldrich, Buchs, Switzerland) was added, the sample was mixed and centrifuged. The upper phase was removed and mixed with 4 mL RLT buffer (Qiagen, Hilden, Germany) and 3 mL ethanol, and processed using the RNeasy Midi Kit (Qiagen, Hilden, Germany). Purified RNA was transported and stored at −80 °C.

### 4.7. RNA Sample Processing and Microarray Data Analysis

A detailed description of RNA sample processing and microarray data analysis can be found in previous publications [13,14,55,56]. Briefly, the Affymetrix GeneChip™ Human Transcriptome Array 2.0 (Affymetrix United Kingdom Ltd., High Wycombe, UK) was used (44,699 protein coding and 22,829 non-protein coding genes). RNA samples with 260/280 nm ratios between 1.97 and 2.04 and RNA integrity numbers > 8.2 were further processed. Fragmented and biotinylated DNA targets were produced from 100 ng of total RNA following the standard Affymetrix GeneChip™ WT PLUS Reagent Kit (Affymetrix United Kingdom Ltd., High Wycombe, UK) protocol. The DNA targets were then hybridized (17 h, 45 °C) on Affymetrix GeneChip™ Human Transcriptome Arrays 2.0. Washing and staining of the chips was performed in the Affymetrix Fluidics Station 450 (Affymetrix United Kingdom Ltd., High Wycombe, UK) according to the standard Affymetrix GeneChip™ Wash, Stain and Scan Kit protocol (Affymetrix United Kingdom Ltd., High Wycombe, UK), and scanned with the Affymetrix 3000 7 G scanner (Affymetrix United Kingdom Ltd., High Wycombe, UK). The Affymetrix Expression Console™ Software (Affymetrix United Kingdom Ltd., High Wycombe, UK) and Transcriptome Analysis Console™ Software (Affymetrix United Kingdom Ltd., High Wycombe, UK) was applied for data analysis. We used the robust multi-array average (RMA) algorithm combined with a quantile normalization of the microarray data [57] where the background noise is subtracted in order to avoid false positive results based on a low signal and high background. Subsequently, a one-way analysis of variance (ANOVA) was employed to determine gene expression differences. The values of two experiment groups were compared and fold changes ≥1.3 or ≤−1.3 with a *p*-value (one-way ANOVA) of <0.05 were considered to represent a significantly differential gene expression. The large number and homogeneity of the applied samples resulted in a low variance within the sample groups and a high sensitivity of the applied method.

### 4.8. Applied Control Strategy for Revealing Microgravity and Hypergravity Sensitivity

To exclude that fold changes measured in the primary microgravity and hypergravity comparisons were due to gravitational or other conditions preceding the respective phase, controls were applied: Intersections were built between the primary comparison and the comparison that represents the previous phase. Transcripts that were shown to be differentially expressed already in the preceding phase were excluded from the pool of microgravity and hypergravity-sensitive Transcripts (see Figure 3a and Figure 4a).

### 4.9. Intra-Platform and Inter-Platform Comparisons

To elucidate if transcripts were either only vag respectively flight-induced microgravity-sensitive, hypergravity-sensitive, or sensitive to both conditions within one experimental platform (intra-platform), intersections between the pools of vag respectively flight-induced microgravity and hypergravity-sensitive transcripts were made. Furthermore, we analyzed if transcripts responded to vag respectively flight-induced microgravity or hypergravity in both experimental platforms by inter-platform intersections.

### 4.10. Functional Annotation Analysis

Compilation of a functional annotation chart was performed with the Functional Annotation Tool DAVID (DAVID Bioinformatics Resources 6.8, NIAID/NIH, https://david.ncifcrf.gov/summary.jsp). As background lists “Homo sapiens” was used, and for compiling the functional annotation chart the GENE-Ontology terms “GOTERM_BP_DIRECT”, “GOTERM_CC_DIRECT”, and “GOTERM_MF_DIRECT" were applied. 

### 4.11. Investigation of Forces Present in a Clinostat

As GBFs fundamentally differ from real microgravity, an analysis of the forces applied on the cells in the vector-averaged gravity environment is warranted. Within the clinostat, the culture medium undergoes a solid body rotation and the cells in suspension are free to move within the medium. The total force acting on the particle results from the contributions of buoyancy and gravity, drag, lift, inertia and added mass. Relevant parameters to consider thus include:

- The cell radius and density: r=5.75 μm and $\rho_{c}=1.05-1.12\;g/\mathrm{mL}$ [58]
- The medium density and viscosity: $\rho_{f}=1.00\;g/\mathrm{mL}$ and μ=1.09−1.14 mPa·s [59]
- The clinostat radius and rotation rate: R=1.5 mm and ω=2π rad/s.

The maximum centrifugal force in this setup is 0.006 g and the characteristic shear Reynolds number, $Re_{s}=\frac{r^{2}\Omega }{\nu }$, is in the order of 10^−4^.

### 4.12. Data Availability

The data sets analyzed for this study are available in the GEO (Gene Expression Omnibus) repository (www.ncbi.nlm.nih.gov/projects/geo), accession no. GSE101102.

## Figures and Tables

**Table 1:** Gravity conditions and controls of experimental groups of the ground based facilities experiments (GBF) and the TEXUS-51 suborbital ballistic rocket flight campaign. Due to the ballistic trajectory, microgravity platforms such as parabolic flights and sounding rockets always contain a hypergravity phase preceding the microgravity phase, limiting the experiments and requiring appropriate controls groups. Additional information on the group is given in brackets. GBF: ground based facilities; TEXUS: German for “Technologische Experimente unter Schwerelosigkeit”; n/a: not available; CC: cell culture control; min: minute(s); H/W: hardware; g: gravity; GC: ground control; BL: baseline; TX: TEXUS-51; hyp-g: hypergravity; s: second(s); max.: maximum; vag: vector-averaged gravity; µg: microgravity; 2D: two dimensional.

		GBF	TEXUS-51
Gravity conditions and controls	**Cell culture control**	**n/a**	**CC** (cell culture control: cells kept under standard cell culture conditions in a cell culture incubator)
	**Hardware 1× *g* ground control**	**1× *g* ground control [5 min]** (sample exposure inside the pipettes used for centrifugation and 2D clinorotation experiments)	**H/W 1× *g* GC** (hardware 1× *g* ground control: ground control samples incubated in the hardware identical in construction to the flight hardware for identification of hardware-based effects)
	**1**×***g*****in-flight control**	**n/a**	**1× *g* IF [5 min]** (1× *g* in flight: sample exposure to 75s hypergravity max. 12.6× *g* plus 5 min 1× *g* centrifugation in the centrifuge on board the suborbital rocket during the flight)
	**Baseline control**	**BL** (filling and draining of pipettes for identification of pipetting effects)	**n/a**
	**Hypergravity**	**9× *g* centrifuge [5 min]** (sample exposure to centrifugal forces of 9g)	**TX hyp-g [75 s, max. 12.6× *g*]** (sample exposure to hypergravity with a maximum of 12.6× *g* during the first 75 s of the rocket launch)
	**Vector-averaged gravity (vag)/Microgravity (µg)**	**2D clinostat [5 min]** (sample exposure to vector averaged gravity)	**TX µg [5 min]** (sample exposure to 75 s, max. 12.6× *g* followed by 5 min microgravity during the suborbital rocket flight)

**Table 2:** Numbers of differentially expressed transcript clusters (TCs) in comparisons of the experiment groups of ground based facilities experiments (GBF). In total 67528 transcript clusters were analyzed by the Affymetrix GeneChip™ Human Transcriptome Array 2.0 including 35219 annotated transcript clusters. Differential expression is defined as *p*-value < 0.05; fold-change ≤ −1.3 or ≥1.3.

Significantly Differentially Expressed TCs in the GBF Experiments					
		1× *g* Control vs. BL	9× *g* Centrifuge vs. 1× *g* Control	2D Clinostat vs. 1× *g* Control	2D Clinostat vs. 9× *g* Centrifuge
All differentially expressed transcript clusters	upregulated	2125	3046	768	339
	downregulated	496	2732	373	623
	total number	2621	5778	1141	962
Annotated differentially expressed transcript clusters	upregulated	890	2089	475	149
	downregulated	187	1238	155	355
	total number	1077	3327	630	504

**Table 3:** Numbers of differentially up and downregulated transcript clusters (TCs) in response to exposure to 2D clinorotation and/or 9× *g* centrifugation. Transcript clusters that were significantly changed in the control comparison 1× *g* control vs. BL were eliminated. Average fold change (FC), minimum and maximum values of each comparison are shown. Differential expression is defined as *p*-value < 0.05; FC ≤ −1.3 or ≥ 1.3. ↑: upregulated TCs; ↓: downregulated TCs. * Refers to the comparison that is mentioned first in the left column. # TCs are regulated in the same direction in 2D clinostat vs. 1× *g* control and 9× *g* centrifuge vs. 1× *g* control comparisons. In contrast, these TCs are regulated reversely in the 2D clinostat vs. 9× *g* centrifuge comparison. -: not applicable.

		Total Number of TCs		Up or Down-Regulated TCs *	Average FC of 9× *g* Centrifuge vs. 1× *g* Control	Min./Max. FC of 9× *g* Centrifuge vs. 1× *g* Control	Average FC of 2D Clinostat vs. 1× *g* Control	Min./Max. FC of 2D Clinostat vs. 1× *g* Control	Average FC of 2D Clinostat vs. 9× *g* Centrifuge	Min./Max. FC of 2D Clinostat vs. 9× *g* Centrifuge
**hypergravity-sensitive transcript clusters** Differentially expressed TCs in 9× *g* centrifuge vs. 1× *g* control	all (annotated)	4596 (2744)	↑	2753 (1945)	1.52 (1.49)	4.28 (2.60)	-	-	-	-
			↓	1843 (799)	−1.44 (−1.43)	−4.00 (−2.39)	-	-	-	-
**vector-averaged gravity-sensitive transcript clusters** Differentially expressed TCs in 2D clinostat vs. 1× *g* control	all (annotated)	871 (495)	**↑**	644 (415)	-	-	1.39 (1.37)	2.38 (1.74)	-	-
			↓	227 (80)	-	-	−1.38 (−1.37)	−2.22 (−1.78)	-	-
**2D clinostat vs. 9× *g* centrifuge-sensitive transcript clusters** Differentially expressed TCs in 2D clinostat vs. 9× *g* centrifuge	all (annotated)	752 (415)	↑	231 (106)	-	-	-	-	1.41 (1.39)	2.43 (1.87)
			↓	521 (309)	-	-	-	-	−1.40 (−1.38)	−2.12 (−1.93)
**hypergravity and vector-averaged gravity-sensitive transcript clusters** Differentially expressed TCs in 9g centrifuge vs. 1× *g* control and in 2D clinostat vs. 1× *g* control	all (annotated)	798 (476)	↑	618 * (409 *)	1.75 (1.71)	4.28 (2.60)	1.40 (1.37)	2.38 (1.74)	-	-
			↓	180 * (67 *)	−1.64 (−1.70)	−2.39 (−2.39)	−1.39 (−1.38)	−2.22 (−1.78)	-	-
**Intersecting set of all three gravity condition comparison groups** Differentially expressed TCs in 2D clinostat vs. 1× *g* control and in 9× *g* centrifuge vs. 1× *g* control and in 2D clinostat vs. 9× *g* centrifuge. #	all (annotated)	222 (148)	↑	199 * (134 *)	2.02 (1.94)	4.28 (2.44)	1.43 (1.4)	2.38 (1.67)	−1.41 (−1.39)	−1.86 (−1.6)
			↓	23 * (14 *)	−2.03 (−2.12)	−2.22 (−2.22)	−1.42 (−1.46)	−1.63 (−1.63)	1.43 (1.46)	1.57 (1.75)

**Table 4:** Numbers of differentially expressed transcript clusters (TCs) in comparisons of the experiment groups of the suborbital ballistic rocket experiment TEXUS-51. In total 67528 transcript clusters were analyzed by the Affymetrix GeneChip™ Human Transcriptome Array 2.0 including 35219 annotated transcript clusters. Differential expression is defined as *p*-value < 0.05; fold change ≤ −1.3 or ≥ 1.3.

Significantly Differentially Expressed TCs in the TEXUS-51 Experiments								
		H/W 1× *g* GC vs. CC	1× *g* IF vs. H/W 1× *g* GC	TX hyp-g vs. H/W 1× *g* GC	TX hyp-g vs. 1× *g* IF	TX µg vs. H/W 1× *g* GC	TX µg vs. 1× *g* IF	TX µg vs. TX hyp-g
All differentially expressed transcript clusters	upregulated	15,995	571	2874	1842	2295	1101	23
	downregulated	7886	536	1144	731	1207	1027	23
	total number	23,881	1107	4018	2573	3502	2128	46
Annotated differentially expressed transcript clusters	upregulated	9243	258	2039	1270	1575	721	5
	downregulated	4828	282	426	193	425	315	2
	total number	14,071	540	2465	1463	2000	1036	7

**Table 5:** Numbers of differentially up and downregulated transcript clusters (TCs) in response to hypergravity and microgravity during the suborbital ballistic rocket flight TEXUS-51. Transcript clusters that were significantly changed in the control comparisons H/W 1× *g* GC vs. CC and 1× *g* IF vs. H/W 1× *g* GC were eliminated. Average fold change (FC), minimum and maximum values of each comparison are shown. Differential expression is defined as *p*-value < 0.05; FC ≤ −1.3 or ≥ 1.3. ↑: upregulated TCs; ↓: downregulated TCs. * Refers to the comparison that is mentioned first in the left column. n/a: no TCs in the respective group. -: not applicable.

		Total Number of TCs		Up or Down-Regulated TCs *	Average FC of TX hyp-g vs. 1× *g* IF	Min./Max. FC of TX hyp-g vs. 1× *g* IF	Average FC of TX µg vs. 1× *g* IF	Min./Max. FC of TX µg vs. 1× *g* IF	Average FC of TX µg vs. TX hyp-g	Min./Max. FC of TX µg vs. TX hyp-g
**hypergravity-sensitive transcript clusters** Differentially expressed TCs in TX hyp-g vs. 1× *g* IF	all (annotated)	346 (112)	↑	104 (56)	1.45 (1.40)	1.94 (1.86)	-	-	-	-
			↓	242 (56)	−1.36 (−1.33)	−1.92 (−1.40)	-	-	-	-
**microgravity-sensitive transcript clusters** Differentially expressed TCs in TX µg vs. 1× *g* IF	all (annotated)	396 (114)	↑	72 (38)	-	-	1.40 (1.39)	1.85 (1.85)	-	-
			↓	324 (76)	-	-	−1.36 (−1.33)	−1.83 (−1.49)	-	-
**TX µg vs. TX hyp-g-sensitive transcript clusters** Differentially expressed TCs in TX µg vs. TX hyp-g	all (annotated)	19 (1)	↑	4 (0)	-	-	-	-	1.37 (n/a)	1.43 (n/a)
			↓	15 (1)	-	-	-	-	−1.39 (−1.33)	−1.68 (−1.33)
**hypergravity and microgravity-sensitive transcript clusters** Differentially expressed TCs in TX hyp-g vs. 1× *g* IF and in TX µg vs. 1× *g* IF	all (annotated)	134 (42)	↑	54 * (31 *)	1.45 (1.44)	1.94 (1.86)	1.42 (1.41)	1.85 (1.85)	-	-
			↓	80 * (11 *)	−1.39 (−1.34)	−1.92 (−1.40)	−1.40 (−1.34)	−1.83 (−1.41)	-	-

**Table 6:** Differentially regulated transcript clusters identified for both, the ground based facilities experiment and the suborbital ballistic rocket experiment TEXUS-51. Comparison of the different data sets revealed 28 annotated hypergravity-sensitive transcript clusters, 5 annotated microgravity-sensitive transcript clusters, and 4 hypergravity and vector-averaged gravity/flight-induced microgravity double-sensitive annotated transcript clusters. Significant differential expression is defined as *p*-value < 0.05; FC ≤ −1.3 or ≥ 1.3.

		Ground Based Facilities Fold Changes		TEXUS-51 Fold Changes	
Gene Symbol	Transcript Cluster ID	9× *g* Centrifuge vs. 1× *g* Control	2D Clinostat vs. 1× *g* Control	TX hyp-g vs. 1× *g* IF	TX µg vs. 1× *g* IF
Hypergravity-sensitive transcript clusters					
*ATP6V1A*	TC03000583.hg.1	1.57		1.34	
*BCAT1*	TC12001309.hg.1	1.31		1.35	
*CAT*	TC11000327.hg.1	1.39		1.31	
*CBFB*	TC16000528.hg.1	1.60		1.42	
*CERS6*	TC02000996.hg.1	1.37		1.42	
*EAF1*	TC03000096.hg.1	1.39		1.30	
*ETS1*	TC11002439.hg.1	1.55		1.38	
*G3BP1*	TC05000844.hg.1	1.54		1.32	
*GAN*	TC16000638.hg.1	1.33		1.35	
*KPNB1*	TC17000611.hg.1	1.93		1.61	
*NET1*	TC10000047.hg.1	1.38		1.45	
*NUDT3*	TC06004129.hg.1	1.68		1.39	
*PAG1*	TC08001361.hg.1	1.32		1.30	
*PELI2*	TC14000332.hg.1	1.43		1.36	
*POM121L9P*	TC05000487.hg.1	1.42		−1.36	
*POM121L9P*	TC05000309.hg.1	1.41		−1.31	
*POM121L9P*	TC05000321.hg.1	1.41		−1.31	
*POM121L9P*	TC06000218.hg.1	1.41		−1.31	
*POM121L9P*	TC06000691.hg.1	1.41		−1.31	
*PRPS1*	TC0X000526.hg.1	1.37		1.31	
*RBM3*	TC0X000255.hg.1	1.35		1.34	
*RBM8A*	TC01001093.hg.1	1.44		1.55	
*RSC1A1; DDI2*	TC01000193.hg.1	1.44		1.32	
*SAMD9*	TC07001605.hg.1	−1.34		1.35	
*SFT2D2*	TC01006289.hg.1	1.72		1.34	
*SRP14*	TC15001228.hg.1	1.42		1.41	
*SRSF6*	TC20000316.hg.1	1.38		1.39	
*TMED8*	TC14001343.hg.1	1.38		1.31	
Vector-averaged gravity/flight-induced microgravity-sensitive transcript clusters					
*G3BP1*	TC05000844.hg.1		1.30		1.30
*KPNB1*	TC17000611.hg.1		1.38		1.53
*NUDT3*	TC06004129.hg.1		1.36		1.45
*POMK; SGK196*	TC08000332.hg.1		1.43		1.32
*SFT2D2*	TC01006289.hg.1		1.39		1.36
Hypergravity and vector-averaged gravity/flight-induced microgravity double-sensitive transcript clusters					
*G3BP1*	TC05000844.hg.1	1.54	1.30	1.32	1.30
*KPNB1*	TC17000611.hg.1	1.93	1.38	1.61	1.53
*NUDT3*	TC06004129.hg.1	1.68	1.36	1.39	1.45
*SFT2D2*	TC01006289.hg.1	1.72	1.39	1.34	1.36

**Table 7:** Functional annotation of the 28 identified annotated hypergravity-sensitive transcript clusters (belonging to 25 genes) listed in Table 6. Eight significant gene ontology enrichments were found. (Microgravity-sensitive transcript clusters and hypergravity and vector-averaged gravity/flight-induced microgravity double-sensitive transcript clusters could not be assigned significantly to any functional annotation).

Category	Enriched Term	Gene Count	% of Genes	Modified Fisher *p*-Value	Fold Enrichment
GOTERM_BP_DIRECT	Transport	5	20	9.9 × 10^−4^	10.5
GOTERM_CC_DIRECT	Cytosol	11	44	4.4 × 10^−3^	2.5
GOTERM_MF_DIRECT	Nucleotide binding	4	16	8.8 × 10^−3^	8.8
GOTERM_MF_DIRECT	Poly(A) RNA binding	6	24	1.1 × 10^−2^	4.1
GOTERM_CC_DIRECT	Nuclear speck	3	12	2.6 × 10^−2^	11.3
GOTERM_MF_DIRECT	RNA binding	4	16	1.9 × 10^−2^	5.6
GOTERM_CC_DIRECT	Intracellular membrane-bounded organelle	4	16	3.2 × 10^−2^	5.4
GOTERM_BP_DIRECT	Regulation of alternative mRNA splicing, via spliceosome	2	8	4.9 × 10^−2^	38.4

**Table 8:** Cross platform and altered gravity condition overlap analysis of transcript cluster (TC) regulation in vector-averaged gravity (vag), flight-induced microgravity, and hypergravity in GBF and TEXUS-51 (the four comparisons 2D clinostat vs. 1× *g* control, TX µg vs. 1× *g* IF, 9× *g* centrifuge vs. 1× *g* control, and TX hyp-g vs. 1× *g* IF are included). A two-step classification of differentially expressed TCs is shown. TCs are first grouped according to a primary comparison shown on the left-hand side (2D clinostat vs. 1× *g* control upregulated, downregulated, non-responsive). The right-hand side of the table shows how the transcripts from these groups respond to (1) the corresponding condition of altered gravity from the other platform, and (2) the contrary altered gravity condition from the other platform.

Sorted by regulation in 2D clinostat vs. 1× *g* control	2D Clinostat vs. 1× *g* Control						TX µg vs. 1× *g* IF				9× *g* Centrifuge vs. 1× *g* Control				TX hyp-g vs. 1× *g* IF			
		All		Annotated			All		Annotated		All		Annotated		All		Annotated	
	up-regulated	181	0.42%	127	0.61%	up-regulated	8	4.4%	5	3.9%	171	94.5%	125	98.4%	8	4.4%	5	3.9%
						down-regulated	2	1.1%	0	0.0%	0	0.0%	0	0.0%	1	0.6%	0	0.0%
						non-responsive	171	94.5%	122	96.1%	10	5.5%	2	1.6%	172	95.0%	122	96.1%
	down-regulated	45	0.11%	12	0.06%	up-regulated	0	0.0%	0	0.0%	0	0.0%	0	0.0%	0	0.0%	0	0.0%
						down-regulated	1	2.2%	0	0.0%	31	68.9%	7	58.3%	0	0.0%	0	0.0%
						non-responsive	44	97.8%	12	100.0%	14	31.1%	5	41.7%	45	100.0%	12	100.0%
	non-responsive	42442	99.47%	20748	99.33%	up-regulated	61	0.1%	32	0.2%	877	2.1%	692	3.3%	82	0.2%	50	0.2%
						down-regulated	295	0.7%	75	0.4%	422	1.0%	159	0.8%	209	0.5%	56	0.3%
						non-responsive	42086	99.2%	20641	99.5%	41143	96.9%	19897	95.9%	42151	99.3%	20642	99.5%
Sorted by regulation in TX µg vs. 1× *g* IF	TX µg vs. 1× *g* IF						2D Clinostat vs. 1× *g* Control				9× *g* Centrifuge vs. 1× *g* Control				TX hyp-g vs. 1× *g* IF			
		All		Annotated			All		Annotated		All		Annotated		All		Annotated	
	up-regulated	69	0.16%	37	0.18%	up-regulated	8	11.6%	5	13.5%	24	34.8%	16	43.2%	51	73.91%	30	81.1%
						down-regulated	0	0.0%	0	0.0%	0	0.0%	0	0.0%	0	0.00%	0	0.0%
						non-responsive	61	88.4%	32	86.5%	45	65.2%	21	56.8%	18	26.09%	7	18.9%
	down-regulated	298	0.70%	75	0.36%	up-regulated	2	0.7%	0	0.0%	3	1.0%	0	0.0%	0	0.00%	0	0.0%
						down-regulated	1	0.3%	0	0.0%	7	2.3%	3	4.0%	65	21.81%	11	14.7%
						non-responsive	295	99.0%	75	100.0%	288	96.6%	72	96.0%	233	78.19%	64	85.3%
	non-responsive	42301	99.14%	20775	99.46%	up-regulated	171	0.4%	122	0.6%	1021	2.4%	801	3.9%	39	0.09%	25	0.1%
						down-regulated	44	0.1%	12	0.1%	446	1.1%	163	0.8%	145	0.34%	45	0.2%
						non-responsive	42086	99.5%	20641	99.4%	40834	96.5%	19811	95.4%	42117	99.57%	20705	99.7%
Sorted by regulation in 9g centrifuge vs. 1× *g* control	9× *g* Centrifuge vs. 1× *g* Control						2D Clinostat vs. 1× *g* Control				TX hyp-g vs. 1× *g* IF				TX µg vs. 1× *g* IF			
		All		Annotated			All		Annotated		All		Annotated		All		Annotated	
	up-regulated	1048	2.46%	817	3.91%	up-regulated	171	16.3%	125	15.3%	29	2.8%	22	2.7%	24	2.3%	16	2.0%
						down-regulated	0	0.0%	0	0.0%	8	0.8%	5	0.6%	3	0.3%	0	0.0%
						non-responsive	877	83.7%	692	84.7%	1011	96.5%	790	96.7%	1021	97.4%	801	98.0%
	down-regulated	453	1.06%	166	0.79%	up-regulated	0	0.0%	0	0.0%	1	0.2%	1	0.6%	0	0.0%	0	0.0%
						down-regulated	31	6.8%	7	4.2%	4	0.9%	0	0.0%	7	1.5%	3	1.8%
						non-responsive	422	93.2%	159	95.8%	448	98.9%	165	99.4%	446	98.5%	163	98.2%
	non-responsive	41167	96.48%	19904	95.29%	up-regulated	10	0.0%	2	0.0%	60	0.1%	32	0.2%	45	0.1%	21	0.1%
						down-regulated	14	0.0%	5	0.0%	198	0.5%	51	0.3%	288	0.7%	72	0.4%
						non-responsive	41143	99.9%	19897	100.0%	40909	99.4%	19821	99.6%	40834	99.2%	19811	99.5%
Sorted by regulation in TX hyp-g vs. 1× *g* IF	TX hyp-g vs. 1× *g* IF						2D Clinostat vs. 1× *g* Control				9× *g* Centrifuge vs. 1× *g* Control				TX µg vs. 1× *g* IF			
		All		Annotated			All		Annotated		All		Annotated		All		Annotated	
	up-regulated	90	0.21%	55	0.26%	up-regulated	8	8.9%	5	9.1%	29	32.2%	22	40.0%	51	56.7%	30	54.5%
						down-regulated	0	0.0%	0	0.0%	1	1.1%	1	1.8%	0	0.0%	0	0.0%
						non-responsive	82	91.1%	50	90.9%	60	66.7%	32	58.2%	39	43.3%	25	45.5%
	down-regulated	210	0.49%	56	0.27%	up-regulated	1	0.5%	0	0.0%	8	3.8%	5	8.9%	0	0.0%	0	0.0%
						down-regulated	0	0.0%	0	0.0%	4	1.9%	0	0.0%	65	31.0%	11	19.6%
						non-responsive	209	99.5%	56	100.0%	198	94.3%	51	91.1%	145	69.0%	45	80.4%
	non-responsive	42368	99.30%	20776	99.47%	up-regulated	172	0.4%	122	0.6%	1011	2.4%	790	3.8%	18	0.0%	7	0.0%
						down-regulated	45	0.1%	12	0.1%	448	1.1%	165	0.8%	233	0.5%	64	0.3%
						non-responsive	42151	99.5%	20642	99.4%	40909	96.6%	19821	95.4%	42117	99.4%	20705	99.7%

**Table 9:** Gene and protein description of hypergravity and microgravity-sensitive transcript clusters differentially expressed in GBFs and sounding rocket flight. Adapted from NCBI and UniProt.

Condition	Gene ID	ENSEMBL Gene Name	UniProt Protein Name	Shortened Description (UniProt)
Hyp-g	*G3BP1*	G3BP stress granule assembly factor 1	Ras GTPase-activating protein-binding protein 1	ATP- and magnesium-dependent helicase that plays an essential role in innate immunity.
Hyp-g & µg	*KPNB1*	karyopherin subunit beta 1	Importin subunit beta-1	Functions in nuclear protein import, either in association with an adapter protein, like an importin-alpha subunit, which binds to nuclear localization signals (NLS) in cargo substrates, or by acting as autonomous nuclear transport receptor.
Hyp-g	*NUDT3*	nudix hydrolase 3	Diphosphoinositol polyphosphate phosphohydrolase 1	Cleaves a beta-phosphate from the diphosphate groups in PP-InsP5 (diphosphoinositol pentakisphosphate) and [PP]2-InsP4 (bisdiphosphoinositol tetrakisphosphate), suggesting that it may play a role in signal transduction.
Hyp-g	*POMK*	protein O-mannose kinase	protein O-mannose kinase	Generates phosphorylated O-mannosyl trisaccharide which is a carbohydrate structure present in alpha-dystroglycan (DAG1), which is required for binding laminin G-like domain-containing extracellular proteins with high affinity.
Hyp-g	*SFT2D2*	SFT2 domain containing 2	vesicle transport protein SFT2B	May be involved in fusion of retrograde transport vesicles derived from an endocytic compartment with the Golgi complex.
µg	*CBFB*	core-binding factor subunit beta	core-binding factor subunit beta	Forms the heterodimeric complex core-binding factor (CBF) with RUNX family proteins. RUNX members modulate the transcription of their target genes, within their regulatory regions via their runt domain, while CBFB is a non-DNA-binding regulatory subunit that allosterically enhances the sequence-specific DNA-binding capacity of RUNX.
µg	*SAMD9*	sterile alpha motif domain containing 9	sterile alpha motif domain-containing protein 9	May play a role in the inflammatory response to tissue injury and the control of extra-osseous calcification, acting as a downstream target of TNF-alpha signaling.
µg	*SRSF6*	serine and arginine rich splicing factor 6	serine/arginine-rich splicing factor 6	Plays a role in constitutive splicing and modulates the selection of alternative splice sites.

## Abbreviations

%	percentage
°C	degree celsius
µg	microgravity
1D	one dimensional
1× *g* IF	1g in flight
2D	two dimensional
3D	three dimensional
BL	baseline
CC	cell culture control
CO_2_	carbon dioxide
DLR	German Aerospace Center
FC	fold change
*g*	gravity
GBF	ground based facility
GC	ground control
GEO	Gene Expression Omnibus
GO	gene ontology
h	hours
H/W	hardware
hyp-g	hypergravity
i.e.	that is
ID	identifier
ISS	International Space Station
km	kilometer
max.	maximum
min	minute
min.	minimum
mL	milliliter
mm	millimeter
mM	millimolar
mPa	millipascale
N	newton
n/a	not available
Pa	pascale
rcf	relative centrifugal force
RPM	random positioning machine
rpm	rounds per minute
RWV	rotating wall vessel
s	second
T	Time after launch
TC	transcript cluster
TEXUS/TX	German: Technologische Experimente unter Schwerelosigkeit
vag	vector-averaged gravity
